# Achieving change in primary care—causes of the evidence to practice gap: systematic reviews of reviews

**DOI:** 10.1186/s13012-016-0396-4

**Published:** 2016-03-22

**Authors:** Rosa Lau, Fiona Stevenson, Bie Nio Ong, Krysia Dziedzic, Shaun Treweek, Sandra Eldridge, Hazel Everitt, Anne Kennedy, Nadeem Qureshi, Anne Rogers, Richard Peacock, Elizabeth Murray

**Affiliations:** 1 eHealth Unit, Research Department of Primary Care and Population Health, University College London, Upper 3rd floor, Royal Free Campus, Rowland Hill Street, London, NW3 2PF UK; 2 Arthritis Research UK Primary Care Centre, Research Institute for Primary Care Sciences and Health Sciences, Keele University, Keele, Staffordshire UK; 3 Health Services Research Unit, University of Aberdeen, Aberdeen, UK; 4 Centre for Primary Care and Public Health, Queen Mary University of London, London, UK; 5 Primary Care and Population Sciences, Faculty of Medicine, University of Southampton, Southampton, UK; 6 Faculty of Health Sciences, NIHR CLAHRC Wessex, University of Southampton, Southampton, UK; 7 Division of Primary Care, University of Nottingham, Nottingham, UK; 8 Archway Healthcare Library, London, UK

**Keywords:** Barriers, Complex interventions, Evidence-based practice, Facilitators, Health services research, Implementation research, Primary care, Systematic review

## Abstract

**Background:**

This study is to identify, summarise and synthesise literature on the causes of the evidence to practice gap for complex interventions in primary care.

**Design:**

This study is a systematic review of reviews.

**Methods:**

MEDLINE, EMBASE, CINAHL, Cochrane Library and PsychINFO were searched, from inception to December 2013. Eligible reviews addressed causes of the evidence to practice gap in primary care in developed countries. Data from included reviews were extracted and synthesised using guidelines for meta-synthesis.

**Results:**

Seventy reviews fulfilled the inclusion criteria and encompassed a wide range of topics, e.g. guideline implementation, integration of new roles, technology implementation, public health and preventative medicine. None of the included papers used the term “cause” or stated an intention to investigate causes at all. A descriptive approach was often used, and the included papers expressed “causes” in terms of “barriers and facilitators” to implementation. We developed a four-level framework covering external context, organisation, professionals and intervention. External contextual factors included policies, incentivisation structures, dominant paradigms, stakeholders’ buy-in, infrastructure and advances in technology. Organisation-related factors included culture, available resources, integration with existing processes, relationships, skill mix and staff involvement. At the level of individual professionals, professional role, underlying philosophy of care and competencies were important. Characteristics of the intervention that impacted on implementation included evidence of benefit, ease of use and adaptability to local circumstances. We postulate that the “fit” between the intervention and the context is critical in determining the success of implementation.

**Conclusions:**

This comprehensive review of reviews summarises current knowledge on the barriers and facilitators to implementation of diverse complex interventions in primary care. To maximise the uptake of complex interventions in primary care, health care professionals and commissioning organisations should consider the range of contextual factors, remaining aware of the dynamic nature of context. Future studies should place an emphasis on describing context and articulating the relationships between the factors identified here.

**Systematic review registration:**

PROSPERO CRD42014009410

**Electronic supplementary material:**

The online version of this article (doi:10.1186/s13012-016-0396-4) contains supplementary material, which is available to authorized users.

## Introduction

Internationally, the pace of change in health care continues to be rapid with a drive to implement more clinically and cost-effective interventions. Policy makers globally recognise the need to speed up the pace and scale of change. In England, the “Innovation Health and Wealth: Accelerating Adoption and Diffusion in the NHS” report published in 2011 set out to support the adoption and diffusion of innovation across the National Health Service (NHS) [1], since reinforced in the 2014 Five Year Forward View [2].

The drive to improve quality of care while reducing costs has led to widespread attempts to promote evidence-based care. There is clearly a room for improvement: a systematic review including 29 guidelines recommendations from 11 studies shows that only one third of the research evidence informing guidelines is being routinely adhered to [3]. Adherence rates vary from just above 20 to over 80 % [3]. Similar findings have been reported across a number of clinical areas in different countries [4–6]. This delay in translation of evidence-based interventions into every day clinical practice is known as the “evidence to practice gap” or “second translational gap” [7].

In the UK, 90 % of health care encounters take place in primary care [8], and in England, primary care has been subject to particularly rapid changes since the Health and Social Care Act of 2012 [9]. Primary care has its own distinctive research and implementation culture, which has been described as contributing to the evidence to practice gap [10]. Primary care organisations vary in characteristics such as team composition, organisational structures, cultures and working practices; and these diverse contexts can make it challenging to implement change. Almost all changes to practice in primary care involve “complex interventions”, i.e. interventions with multiple interconnecting components [11], and complex interventions can be particularly hard to implement, as they are likely to require change at multiple levels.

A “meta-review” or systematic review of reviews was judged to be the most appropriate method to address this complex area as there is a vast literature which is highly heterogeneous [12, 13]. Existing reviews tend to focus either on a particular type of complex intervention (e.g. introduction of new technologies [14] or promoting uptake and use of guidelines [15]) or on a particular health condition (e.g. mental health [16] or diabetes [17]). Conducting a systematic review of reviews enables the findings of individual reviews to be brought together, compared and contrasted, with the aim of providing a single comprehensive overview, which can serve as a simple introduction to the challenges of achieving change and implementing complex interventions in primary care for managers, clinicians or policy makers.

In this review of reviews, we aim to identify, summarise and synthesise the available review literature on causes of the evidence to practice gap, referred to as any given explanation(s) of why and how complex interventions fail to be implemented in clinical practice, in the primary care setting.

## Methods

### Search strategy

A comprehensive search was carried out in five electronic databases (including MEDLINE, EMBASE, CINAHL, Cochrane Database of Systematic Reviews and PsychINFO) to seek all potentially eligible papers. The search was performed by the primary reviewer (RL), supported by a specialist librarian (RP). The search strategy was developed using both medical subject headings (MeSH), for example: “translational medical research”, “evidence-based practice”, “general practice”, “review”, “review literature as topic” and free-text words, for example, evidence to practice, evidence-practice gap, family doctor, implementation, adoption and barriers. Articles reported in English and published up to December 2013 were eligible for inclusion in this review. Citation searches were carried out in ISI Web of Science and reference lists of all included articles were screened for additional literature. Details of the search strategy for MEDLINE (Ovid) are provided in Additional file 1.

### Eligibility criteria

Eligibility criteria were defined to enable transparent and reproducible selection of papers for inclusion. The following a priori definitions were applied:

Primary Care in developed countries: the Royal College of General Practitioners (RCGP) has defined primary care as “the first level contact with people taking action to improve health in a community” [18]. Primary care teams are defined as teams or groups of health professionals that include a primary care physician (i.e. general practitioners, family physicians, nurse practitioners and other generalist physicians working in primary care settings). Developed countries are often referred to as more economically developed countries, and a list of high-income member countries has been provided by the Organisation for Economic Co-operation and Development (OECD) [19]. We included reviews with at least 50 % original studies from “primary care” in developed countries. Reviews exclusively on dental practices, pharmacies or developing countries were excluded.

Complex interventions: defined as interventions with several interconnecting components that operate at multiple levels [11].

Implementation: defined as all activities that occur between making an adoption commitment and the time that an innovation becomes part of the organisational routine, ceases to be new or is abandoned [20].

Review: any type of review that provided a description of methods (e.g. identification of relevant studies, synthesis), such as systematic reviews (structured search of bibliographic and other databases to identify relevant literature; use of transparent methodological criteria; presentation of rigorous conclusions about outcomes), narrative reviews (purposive sampling of the literature use of theoretical or topical criteria to include papers on the basis of type, relevance and perceived significance, with the aim of summarising, discussing and critiquing conclusions) and meta-syntheses using definitions provided by Mair et al. [13].

To be included, a paper had to be a review of the causes of the evidence to practice gap for complex interventions in primary care. As our primary focus was professional behaviour change, we excluded reviews that only examined patient behaviours.

### Study selection

Duplicate references were deleted and titles, and abstracts of all the records obtained from the search were independently double-screened. The primary review author, RL, screened all identified citations (titles and abstracts) for potential inclusion; co-authors acted as the second reviewers. In the first instance, a sample of 20 % of citations was screened by RL and other authors (~100 citations each). Following this, the group met and had an in-depth discussion to resolve any uncertainty or disagreement about applying the inclusion/exclusion criteria before screening the remaining citations. RL obtained the full text of potentially eligible articles which were assessed for eligibility against the pre-specified inclusion and exclusion criteria by two reviewers (RL, EM) working independently. Any discordance or uncertainty was resolved through discussion between the two reviewers initially and the involvement of a third reviewer as necessary. Reasons for exclusion were recorded and are presented in the Preferred Reporting Items for Systematic Reviews and Meta-analyses (PRISMA) flow diagram [21].

### Data extraction

For all eligible full text articles, data were extracted by a single reviewer (RL) using standardised structured data abstraction forms. The content of the data abstraction forms were reviewed for validity by the co-authors with extensive experience in systematic review methodologies and implementation/evaluation of complex interventions, to ensure all key information from the included reviews were captured. Data extracted included the following: author, year, title, objective, setting, eligibility criteria for selecting studies, synthesis method, number of and design of included primary studies, use of theoretical framework(s). Data extraction was checked by co-authors for a sample of 25 % of all included reviews, using a quality assurance form. The papers were randomly selected from each review topic or category (e.g. guideline, technology, prescribing behaviour) (Additional file 2) to ensure same level of quality assurance was carried out in all review categories.

For this review, as we aimed to synthesise a body of qualitative literature and not determine an effect size, we did not undertake a formal quality appraisal of the included reviews [22]. However, we have described the degree to which each included review conformed with the PRISMA checklist [21].

### Data synthesis

Data were synthesised using principles of meta-ethnography [23, 24], based on an iterative, interpretive and inductive approach. Meta-ethnography rests on the reviewers’ interpretation of the findings, which may include themes, categories and relationships, arising from the data of the original findings, to produce new interpretations that incorporate the meanings of the included studies [25].

The first stage seeks to determine how the studies are related; this can be achieved by creating a list of initial themes or concepts used in each account. Initially, we extracted key information and concepts from results and discussions of the included reviews; this included the main themes related to the causes of the evidence to practice gap. Data from discussions were extracted because they often contained further interpretations from the reviewers, which provided important insights. Attempts were made to differentiate between interpretations made by the original authors based on the primary data and those made by the authors of the reviews, although this was not always possible.

The second stage involves translating the studies into one another (comparisons between studies with regards to key themes/concepts) [26]. This process allowed the identification of common and recurring elements (or translation of the results of the papers into a common form) in the literature by reading the reviews again, taking into account the extracted data, and grouping similar concepts in the extraction grid as themes [23]. These themes formed columns of the grid, and a row for each review was created. The construction of this grid allowed the relationships between themes and between reviews to be explored. A pilot synthesis was carried out using a sample of 20 papers which was reviewed and discussed extensively by the authors, before undertaking further analysis. To preserve the meaning of the included studies, the terminology used in each review was maintained within the grid. Each theme was carefully defined (also known as descriptors) to facilitate coding, by the primary author (RL) with input from all authors. The list of descriptors was reviewed repeatedly by the authors and refined. Data were re-categorised from one construct to another, and some constructs were refined and re-configured if necessary [23]. Any uncertainty about coding was discussed between RL, EM, FS and BNO. When each concept from the reviews had been translated into the grid, all the authors examined and commented on the themes and data within the grid to ensure all data were coded into appropriate constructs, and a final version was agreed. Following such iterative and rigorous process of data synthesis, 25 % of included reviews (randomly selected from each review topic or category) were double-coded by the co-authors using a quality assurance form.

The third stage involves synthesising translations which include three main forms of synthesis: reciprocal (concepts are common and recurring); refutational (concepts are conflicting across included reviews); and line of argument where an overarching narrative is developed that summarises and represents the key findings of the included reviews [23]. Following the review of the grid (mapping of data onto the constructs), the authors collectively agreed that the relationships between included reviews appeared to be reciprocal, with many common themes occurring across studies and from which a line of argument could be constructed. The line of argument synthesis is described in the “Results” section, presented in the form of a conceptual framework and also in the “Discussion” section where the interpretations of the data are discussed and implications for clinical practice and future research are described.

This systematic review is reported in accordance with the ENTREQ statement guidelines to enhance transparency in reporting the synthesis of qualitative research (see Additional file 3) [27]. The full version of the review protocol was published elsewhere [22]. This systematic review was part of a NIHR SPCR funded project (SPCR FR4 project number: 122). The systematic review protocol was registered on the PROSPERO database (CRD42014009410).

## Results

### Identification of relevant reviews

**Fig. 1:**
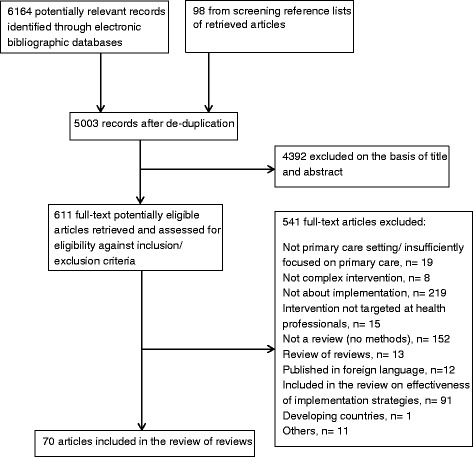
PRISMA flow diagram

Searches of the five electronic databases to December 2013 yielded a total of 6164 potentially eligible papers. After screening of titles, abstracts and full text papers, 70 reviews were included. Figure 1 presents the PRISMA flow diagram of study selection.

### Characteristics of included reviews

None of the included papers used the term “cause” or intended to investigate causes of the second translational gap. It quickly became apparent that a descriptive approach prevailed with the included papers expressing “causes” in terms of “barriers and facilitators” to implementation; hence, we adopted this approach despite being aware of the criticisms of this in the literature [28]. Of the 70 included papers, 64 reported barriers, 49 reported facilitators, and 46 reported both. Reviews encompassed a wide range of different topic domains: 13 reviews focused on research evidence/guideline implementation, 11 on quality of care and disease management, 26 on technology based intervention implementation, 12 on public health and prevention programmes, 6 on role integration/collaborative working, 1 on prescribing and 2 on others. Details of how topics were categorised are described in Additional file 2. Thirty-two reviews (46 %) included original studies from primary care only, with the rest including studies from mixed health care settings.

Eighteen reviews (26 %) were undertaken in the United States of America (USA), 16 (23 %) in Canada, 15 (21 %) in the UK, 8 (11 %) in Australia and 10 (14 %) in Europe. The number of original studies included in the reviews ranged from 2 to 225. The primary studies included in the reviews had been undertaken in developed countries worldwide, with 17 reviews stating that the original studies were predominantly conducted in the USA. Seventeen reviews included only quantitative studies, 4 included only qualitative studies and 30 included both quantitative (e.g. survey) and qualitative studies. Data came from multiple perspectives including health care professionals and administrative staff. Details of included reviews can be found in Table 1.

**Table 1:** Characteristics of included reviews

First author, year (reference) Title Review type	Aims and objectives	Inclusion and exclusion criteria	• Number and type of included studies • Description of study screening and abstraction process • Description of study selection or flow diagram?	• Synthesis method • Quality assessment? Any rating or commentary? • Theory used/considered? • Perspective(s)	Barriers/facilitators/both
**Guideline implementation and evidence-based practice**					
Novins DK 2013 [15] Dissemination and implementation of evidence-based practices (EBPs) for child and adolescent mental health: a systematic review Systematic review	To identify key findings from empirical studies examining the dissemination and implementation of EBPs for child and adolescent mental health	Inclusion criteria Included were English language empirical journal articles that examined the dissemination and implementation of EBPs in child and adolescent mental health between 1991 and December 2011	• 60 (quantitative and qualitative) • Yes • Yes	• Framework analysis • Yes • Yes (analysis) EPIS model/framework and CFIR • Unclear	Facilitators only
Zwolsman S 2012 [66] Barriers to GP’s use of evidence-based medicine: a systematic review Systematic review	To determine the barriers encountered by GPs in the practice of evidence-based medicine and to come up with solutions to the barriers identified	Inclusion criteria Studies about barriers in the practice of evidence-based medicine (EBM); studies with GP as subjects; reported outcomes, barriers to the practice of evidence-based medicine/more than one of the EBM steps. Exclusion criteria Studies that had primary care physicians as subjects and in which the outcomes of GPs were not presented separately. Studies describing the application or use of specific guidelines	• 22 (9 qualitative, 12 quantitative and one mixed methods) • Yes • Yes	• Analysis based on Model of evidence-based decision-making in GPs • Yes (criteria used by another similar review on EBM) • No • GPs	Barriers only
Mickan S 2011 [3] Patterns of ‘leakage’ in the utilisation of clinical guidelines: a systematic review Systematic review	To review evidence in different settings on the patterns of ‘leakage’ in the utilisation of clinical guidelines using Pathman’s awareness-to-adherence model. To summarise any identified barriers to guideline implementation	Inclusion criteria Studies that look at the utilisation of one or more clinical practice guideline recommendation(s), that measure awareness and agreement and either adoption or adherence (or both); Design: any primary survey or cross-sectional study; Response rate: not specified as we wished to include internet surveys, and determining the denominator is not always possible; Outcome measures: both objective and self-reported Specialty or area: any area of health care Health care objective: any (e.g. diagnosis, prevention, screening)	• 11 surveys (8 mailed surveys, 2 internet surveys, 1 was given to participants after a personal interview) • Clearly stated • Yes	• Unclear • Yes (using a proforma quality criteria) • Yes (Pathman’s awareness-to-adherence model) • Physicians	Facilitators and barriers
Ogundele M 2011 [52] Challenge of introducing evidence-based medicine into clinical practice: an example of local initiatives in paediatrics	To review the available literature on how clinicians meet the daily challenge of translating medical information into clinical evidence-based medicine	Inclusion criteria Unclear Exclusion criteria None stated	• Unclear • Unclear • Unclear	• Narrative • No • No • Professionals	Facilitators and barriers
Lineker SC 2010 [100] Educational interventions for implementation of arthritis clinical practice guidelines in primary care: effects on health professional behaviour Systematic review	To evaluate the influence of educational programmes designed to implement clinical practice guideline for osteoarthritis and rheumatoid arthritis in primary care	Inclusion criteria English articles published between 1994 and 2009 and were related to implementation of arthritis CPG in primary care; prospective evaluation studies that targeted primary care providers working with adults with rheumatoid arthritis or osteoarthritis and if they reported behavioural outcomes that ensured actual knowledge utilisation in primary care Exclusion criteria None stated	• 7 (6 randomised controlled trials (RCTs) and 1 before and after study) • Unclear • No	• Narrative • Yes (Modified Philadelphia Panel grading system) • No • GPs	Barriers only (not stated as an objective; data found in results and discussion)
Kendall E 2009 [68] When guidelines need guidance considerations and strategies for improving the adoption of chronic disease evidence by general practitioners Literature review	To investigate barriers to guideline uptake and dissemination practices and options for improving the process of embedding evidence into practice	Inclusion criteria Peer-reviewed journals between January and April 2008 Studies that explored the barriers and issues associated with the use of guidelines in general practice Exclusion criteria Unclear	• Unclear • Not stated • Not given	• Unclear • No • Yes (discussion) Uptake model • GPs	Facilitators and barriers
Langberg JM 2009 [59] Interventions to promote the evidence-based care of children with attention deficit-hyperactivity disorder (ADHD) in primary-care settings Review	To review the efficacy of intervention models that designed to improve physician use of the evidence-based recommendation for evaluating and treating children with ADHD	Inclusion criteria Interventions that specifically target the improvement of evidence-based ADHD-related physician practice behaviours, and not mental health care in general and only intervention that published quantitative outcomes were included Exclusion criteria School and community based approaches for improving the identification and management of children with ADHD that have been proposed but not evaluated formally	• 9 (2 observational, 1 RCT, 1 cluster RCT, 5 interrupted time series) • Not stated • Not given	• Unclear • No (quality not discussed) • No • Physicians	Facilitators and barriers (not stated as an objective)
Dulko D 2007 [35] Audit and feedback as a clinical practice guideline implementation strategy: a model for acute care nurse practitioners Systematic review	To evaluate the effectiveness of audit and feedback as a guideline implementation strategy	Inclusion criteria Articles published in English between 2001 and 2005; focused on physical symptoms related to cancer or cancer treatment Exclusion criteria None stated	• 16 (unclear) • Not stated • Not given	• No • No • Yes (discussion) Change theory • Nurse practitioners	Facilitators and barriers
McKenna H 2004 [38] Barriers to evidence-based practice in primary care: a review of the literature Narrative review	To examine evidence-based practice in primary and review the barriers encountered by professionals when attempting to introduce evidence into practice	Inclusion criteria Articles related to terms such as primary care, barriers to research utilisation and evidence-based practice and those that focus on policy and research papers, the role of patients and client in the planning and delivery of primary care Exclusion criteria None stated	• Unclear • Not stated • Not given	• Narrative • No • Yes (discussion) Kitson’s conceptual framework enabling implementation of evidence-based practice • Health professionals	Facilitators and barriers
Parsons J 2003 [75] Evidence-based practice in rural and remote clinical practice: where is the evidence? Systematic review	To review the evidence regarding barriers to implementing research findings in rural and remote settings	Inclusion criteria Articles that included information on the barriers to the implementation of evidence faced in rural and remote areas; interventions for implementing evidence-based practice or an element of evidence-based practice in rural and remote areas Exclusion criteria –	• 2 (survey) • Not stated • Not given	• Narrative • Quality of the included studies and their applicability were discussed • No • Health professionals	Barriers only
Cabana MD 1999 [39] Why don’t physicians follow clinical practice guidelines? A framework for improvement Systematic review	To review barriers to physician adherence to clinical practice guidelines To examine candidate titles of papers describing theories of physician behaviour change to find constructs useful in describing barriers	Inclusion criteria Articles that focused on clinical practice guidelines, practice, parameters, clinical policies, national recommendations or consensus statements, and that examined at least 1 barrier to adherence. Only barriers that could be changed by an intervention were included. Exclusion criteria None	• 76 (surveys and qualitative studies) • Yes • Yes	• Theory based analysis • No (quality was discussed) • Yes (analysis) The knowledge, attitudes, behaviour framework • Physicians	Barriers only
Wensing M 1998 [83] Implementing guidelines and innovations in general practice: which interventions are effective? Systematic review	To evaluate the effectiveness of interventions in influencing the implementation of guidelines and adoption of innovations in general practice	Inclusion criteria Studies were included if one or more interventions were used to improve professional behaviour in general practice and if the effect on actual behaviour was measured RCTs, controlled trials, controlled before and after studies Exclusion criteria Non-randomised controlled trials that did not perform pre-intervention measurement in intervention or control group	• 61 “best evidence” studies (143 studies identified) (quantitative) • Yes • Yes	• Narrative • Yes (no checklist was used—selection of “best evidence” studies were made) • No • Unclear	Barriers only (in discussion; quality not relevant)
Davis AD 1997 [89] Translating guidelines into practice. A systematic review of theoretic concepts, practical experience and research evidence in the adoption of clinical practice guidelines (CPG) Systematic review	To explore the variables affecting physicians’ adoption of clinical practice guidelines and describe outcomes of trials of educational interventions to change physicians’ behaviour or health care outcomes	Inclusion criteria Studies of CPG implementation strategies and reviews of such studies were selected	• Unclear • No • No	• Descriptive/narrative • No • No • Professionals	Facilitators and barriers
Grilli R 1994 [88] Evaluating the message: the relationship between compliance rate and the subject of a practice guideline Literature review	To explore the relationship between providers’ compliance and some key aspects of the clinical messages in practice guidelines	Inclusion criteria Papers had to present compliance rates with practice guidelines developed by official organisations and had to target providers as the audience	• 23 • No • No	• Narrative • No • Yes (diffusion of innovation mentioned in the introduction) • Physicians	Barriers only
*Management of care*					
Lovell A 2014 [101] Advanced care planning (ACP) in palliative care: a systematic literature review of the contextual factors influencing its uptake 2008–2012 Systematic review	To identify the contextual factors influencing the uptake of Advanced care planning in palliative care	Inclusion criteria Only primary research reporting on ACP within palliative care was included. Studies on the views of organisations involved in aged and end of life care were also included Exclusion criteria Studies that evaluated a novel intervention, tool or model of ACP were excluded	• 27 (half or 13 included studies used qualitative methodology; 3× mixed methods; 11× quantitative methods) (10 studies conducted in USA, UK 8, Australia 4, Belgium 2, Netherlands 1, China and Taiwan 2) • Yes • Yes	• Thematic synthesis • Yes (NICE quality appraisal checklist) Quality of the studies varied. Few based their work on explicit theoretical frameworks PRISMA checklist was used to conduct this review • No • Primary care health care professionals	Facilitators and barriers
Holm AL 2012 [71] Chronic care model (CCM) for the management of depression: synthesis of barriers to and facilitators of success Systematic review	To identify barriers to, and facilitators of success when implementing the CCM for the management of depression in primary care	Inclusion criteria Published in English, implementation or use of the CCM, and primary care and depression as one of the chronic illnesses covered Exclusion criteria Not using CCM, chronic illnesses not including depression, and reviews (also studies published in books and dissertations)	• 13 (quantitative and qualitative) • Unclear • Yes	• Thematic analysis • Yes (adapted a framework from both quantitative and qualitative research traditions; quantitative: sample size, reliability, validity, and transferability. Qualitative: trustworthiness, credibility, confirmability, dependability and transferrability • No • Professionals and administrative staff	Facilitators and barriers
Sales AE 2012 [102] The use of data for process and quality improvement in long-term care and home care: a systematic review of the literature Systematic review	To determine how the resident assessment instrument minimum data set (RAI) have been used in process or quality improvement activities in the continuing care sector	Inclusion criteria Discussed continuing care in a long-term care and health care setting; involved some form of intervention relating to quality or process improvement, and used RAI data in the quality or process improvement intervention.	• 24 (quantitative) • Yes • Yes	• Descriptive/narrative • No • No • Unclear	Barriers only (in discussion; quality not relevant)
Zhang J 2012 [103] System barriers associated with diabetes management in primary care Systematic review	To explore system barriers to diabetes management in primary care and solutions that overcome the system barriers and the role of nurse practitioners in addressing these system barriers	Inclusion criteria English only articles and articles specifically focused on system barriers for diabetes management in primary care settings were included Exclusion criteria None stated	• 31 (both systematic reviews and primary studies) • Not stated • Not given	• Unclear • Not stated • No • Unclear	Facilitators and (largely) barriers
Hoare K 2012 [53] The role of government policy in supporting nurse-led care in general practice in the UK, New Zealand and Australia: an adapted realist review Systematic review and realist review	Realist review to examine the theory that clinical governance was the main driver to stimulate practice nurse development To examine the role of government policy in primary care and its association with nurse-led care in the UK, New Zealand and Australia between 1998 and 2009	Inclusion criteria Systematic review—the study had to report primary research involving practice nurses or demographical statistics of nurse-led clinics in general practice	• 45 (mixed study types including policy documents) • Yes • Yes	• Realist synthesis • Realist synthesis—the reviewer reads the paper to search for evidence that may support the initial theory and so contribute to fuller development of an explanatory model. No quality assessment tools were suitable for the systematic review • No • Unclear	Facilitators and barriers
Nam S 2011 [78] Barriers to diabetes management: patient and provider factors Systematic review	To summarise existing knowledge regarding various barriers of diabetes management from the perspectives of both patients and clinicians	Inclusion criteria Cross-sectional studies, RCTs, observational studies and qualitative studies. Studies had to be relevant to type 2 diabetes or patient and health care providers’ barriers to diabetes management Exclusion criteria Review articles and epidemiological studies were largely excluded, unless they were directly relevant to the themes that were part of this review	• 80 • Not stated • Not given	• Narrative synthesis • No • No • Clinicians	Barriers
Addington D 2010 [16] Facilitators and barriers to implementing quality measurement in primary mental health care Systematic review	To identify facilitators and barriers to implementing quality measurement in primary mental health care	Inclusion criteria The study need to focus on primary care and refer to a quality improvement tool, or the process of implementing quality measurement, quality indicator, or quality improvement Exclusion criteria None	• 57 (qualitative case studies, interviews, RCTs, focus groups, cross-sectional qualitative/quantitative surveys, quasi-experimental studies, prospective cohorts, cluster analyses, controlled before and after trials, audits) • Yes • Yes	• Content analysis; descriptive • No • No • GPs, nurses and administrative staff	Facilitators and barriers
Koch T 2010 [79] Rapid appraisal of barriers to the diagnosis and management of patients with dementia in primary care: a systematic review Systematic review	To systematically investigate current evidence about the barriers to dementia diagnosis in primary care	Inclusion criteria Studies related to barriers to the recognition of dementia. Exclusion criteria Studies about pharmacological interventions (for dementia or Alzheimer’s disease), studies related to the validity or usefulness of specific cognitive function tests, studies not related to primary care setting, clinical discussion about dementia diagnoses or care, letters, publications in languages other than English	• 11 (6 qualitative, 3 quantitative, 2 mixed methods) • Yes • Yes	• Thematic analysis • No • No • Primary care physicians	Facilitators and barriers
Zwar N 2006 [104] A systematic review of chronic disease management Systematic review	To investigate the facilitators and barriers to effective interventions for chronic disease in primary health care (one of the three research questions)	Inclusion criteria Systematic reviews, RCTs, controlled clinical trials, controlled before-and-after studies and interrupted time series studies involving adults aged 18 years and over with one or more of the following chronic conditions: hypertension, coronary heart disease, type 2 diabetes, lipid disorders, asthma, chronic obstructive pulmonary disease, arthritis and osteoporosis Exclusion criteria Studies published before 1990, in a language other than English or pertaining only to a change in patient knowledge	• 141 studies and 23 systematic reviews • Yes • Yes	• Narrative • Yes (Joanna Brigg’s institute and EPOC criteria) • Yes • Unclear	Facilitators and barriers
Johnston G 2000 [67] Reviewing audit: barriers and facilitating factors for effective clinical audit Literature review	To assess the main facilitators and barriers to conducting the audit process	Inclusion criteria Papers which addressed empirical evidence from studies of clinicians’ views, and also theoretical discussions were included in this study. Exclusion criteria –	• 93 (qualitative only) • Yes • Yes (flow chart not given)	• Thematic analysis • No • No • Professionals and managers	Facilitators and barriers
Renders CM 2001 [87] Interventions to improve the management of diabetes mellitus in primary care, outpatient and community settings (Cochrane review) Systematic review	To examine the effectiveness of different interventions, targeted at health professionals or the structure in which they deliver care To determine which intervention strategy or parts of intervention strategies are the most effective and what do they have in common	Inclusion criteria Population—health care professionals (including physicians, nurses, pharmacists) taking care of non-hospitalised patients with type I or II diabetes in primary care, outpatients and community settings Type of interventions—organisational, professional and financial interventions; patient oriented interventions that included alongside professional and organisational interventions (all compared to usual care) Exclusion criteria Solely patient oriented interventions including patient education, mail order pharmacies, consumer participation in health care organisation	• 41 (RCTs, controlled before and after studies, interrupted time series) Outcomes: Health professional performance, e.g. blood markers, making a follow-up, referral, exam of the feet Patient outcomes, e.g. cardiovascular risk factors, hospital admissions, mortality, no. of complications Self-report subjective measures, e.g. patient/provider satisfaction, quality of life) • Yes • Yes	• Narrative • Yes (EPOC checklist/quality criteria) • No • Unclear (barriers not main objective)	Barriers only
*E-health technology*					
Gagnon MP 2014 [14] Barriers and facilitators to implementing electronic prescribing: a systematic review of user groups’ perceptions Systematic review	To identify user groups’ perceptions of barriers and facilitators to implementing electronic prescription (e-prescribing) in primary care	Inclusion criteria Studies with an empirical design, either qualitative, quantitative, or mixed methods. Studies should present a clearly stated data collection process as well as research methods and measurement tools used. Studies focused on the users’ (physicians, clinical staff, nurses, pharmacists, pharmacy staff and others such as patients IT staff and managers) experience of e-prescribing implementation Primary care, including ambulatory or community health care settings. Studies had to provide data on barriers and facilitators to e-prescribing implementation in their results or discussion sections to be included Exclusion criteria Editorials, comments, position papers, unstructured observations	• 34 publications (28 individual studies) Surveys (42.9 %; *n* = 12) and qualitative methods (39.9 %; *n* = 11); mixed methods (17.9 %; *n* = 5) >1/3 of the studies (35.7 %) included a theoretical framework 12 studies (42.9 %) exclusively involved physicians, 2 studies targeted exclusively pharmacists, 6 studies included physicians and their staff, 3 studies involved pharmacists and their staff, 5 studies include more than one of these groups • Yes • Yes	• Use of logical model of health care quality proposed by Donabedian, coupled to the themes proposed by Barber et al.) • Yes. Mixed methods appraisal tool (MMAT) • Yes. Data extraction developed used both inductive and deductive methods, following theoretical concepts like the technology acceptance model and the diffusion of innovations theory • Professionals and staff	Facilitators and barriers
Hage 2013 [94] Implementation factors and their effect on e-health service adoption in rural communities: a systematic literature review Systematic review	To contribute our understanding of the implementation factors that determine successful e-health adoption in rural communities	Inclusion criteria Papers focused on rural context, implementation, e-health content, adoption outcomes Empirical studies addressing implementation published in peer-reviewed journals. Papers were written in English	• 51 (26 quantitative approach, 14 qualitative, 11 mixed approach) • Yes • Yes	• See below • Yes (two checklists used) • Use of a theoretical framework for analysis (context, process, content, adoption outcomes) • Unclear	Facilitators and barriers
Lau F 2012 [58] Impact of electronic medical record on physician practice in office settings: a systematic review Systematic review	To examine the impact of electronic medical records (EMR) in the physician office, factors that influenced their success and the lessons learned	Inclusion criteria Studies that were published in English, evaluated use of an EMR in an office-based setting, were based on original data, had physicians as primary end users, focused on clinical functions, reported impact on practice performance, patient outcomes, or physician-patient interactions Exclusion criteria Studies were excluded if their EMRs were part of the hospital information systems or were hospital ambulatory clinic settings or if there were only survey studies	• 43 (27 controlled and 16 descriptive studies) • Yes • Yes	• Use of the Clinical Adoption Framework as a conceptual scheme • No • Yes • Physicians	Factors
Gagnon MP 2012 [31] Systematic review of factors influencing the adoption of information and communication technologies (ICT) by health care professionals Systematic review	To review factors that are positively or negatively associated with ICT adoption by health care professionals in clinical settings	Inclusion criteria Qualitative, quantitative, or mixed method methodology used to collect original data was described; the intervention for promoting the adoption or the use of a specific ICT in health care settings was described; the outcomes measured included barriers and/or facilitators to the adoption of a specific ICT application by health care professional, including professionals in training. Studies reported in French, English or Spanish	• 101 (quantitative and qualitative) • Yes • Yes	• Narrative synthesis using inductive and deductive methods • Yes (Pluye mixed methods review scoring checklist) • Yes • Professionals (physicians and nurses)	Facilitators and barriers
Pereira JA 2012 [72] Barriers to the use of reminder/recall (RR) interventions for immunizations: a systematic review Systematic review	To identify providers’ perceived barriers to use of reminder/recall measures to address patient under-immunisation and improve coverage	Inclusion criteria Studies that examined the perceptions of health care providers regarding barriers towards implementing either provider-directed RR or patient-directed RR interventions for childhood and/or adult immunisations Surveys, focus groups or interviews. English; contained original data, and described studies using quantitative and/or qualitative methodologies Exclusion criteria Reviews, editorials, commentaries, and practice guidelines, conference abstracts	• 10 (perceptions of family physicians, nurse practitioners, paediatricians, and other immunisation staff) (5 surveys, 1 interview, 2 focus groups, 2 mixed methods) • Yes • Yes	• Thematic analysis • Yes (CASP) all studies were moderate-high quality • No • Professionals and staff (family physicians, nurses, administrators)	Barriers only
Saliba V 2012 [105] Telemedicine across borders: a systematic review of factors that hinder or support implementation Systematic review	To systematically identify factors that hinder or support implementation of cross-border telemedicine services worldwide in the last two decades	Inclusion criteria Studies which described the use of telemedicine to deliver cross-border health care and described the factors that hinder or support implementation of cross-border telemedicine services All study designs	• 94 (quantitative and qualitative) • Yes • Yes	• Narrative synthesis (using adapted framework developed by a project for the economic and social research council methods programme) • Yes • Yes • Unclear	Facilitators and barriers
Fontaine P 2010 [51] Systematic review of health information exchange (HIE) in primary care practices Systematic review	A systematic review of literature related to the adoption of HIE by ambulatory and primary care practices, with an emphasis on benefits, barriers and the overall value to the practice	Inclusion criteria The content dealt with electronic HIE in the US; the HIE involved at least one stakeholder in an ambulatory office or primary care practice, or described benefits, barriers or concerns relevant to ambulatory practices	• 64 (quantitative and qualitative) • Yes • Yes	• Themes emerged from the publications • No • No • Primary care professionals	Facilitators and barriers
Ludwick DA 2009 [77] Adopting electronic medical records in primary care: Lessons learned from health information systems implementation experience in seven countries Systematic review	To identify the current state of knowledge about health information systems (HIS) adoption in primary care To understand factors and influencers affecting implementation outcomes from previous HIS implementations experiences	Inclusion criteria Peer-reviewed and grey literature published during the period 2000 to the end of 2007 from Canada, the USA, Denmark, Sweden, Australia, New Zealand and the UK; articles about implementation of health informatics systems Exclusion criteria None stated	• 86 (study types unknown) • Yes • Yes	• Narrative • No • Yes (socio-technical perspective) • Users including physicians	Facilitators and barriers
Mollon B 2009 [74] Features predicting the success of computerised decision support system (CDSS) for prescribing: a systematic review of randomised controlled trials Systematic review	To determine which features of system design or implementation were associated with the success or failure of prescribing (Rx) CDSS implementation, change in provider behaviour, and change in patient outcomes	Inclusion criteria Reports of RCTs of prescribing CDSS published in English. They only considered systems which intervened before a drug therapy had been chosen by a physician or had the ability to suggest alternate therapies to be a RxCDSS Outcomes: implementation, change in provider behaviour, and change in patient outcomes Exclusion criteria Systems whose sole purpose was to offer ‘fine tuning’ advice on a pre-defined therapy, usually dose modification were not included. Systems primarily focused on diagnosis, vaccination, or nutrition were also excluded	• 41 (quantitative) • Yes • Yes	• Narrative • Yes (modified scale adapted from Garg et al.) • No • Unclear	Facilitators only
Waller R 2009 [106] Barriers to the uptake of computerised cognitive behavioural therapy (cCBT): a systematic review of the quantitative and qualitative evidence Systematic review	To systematically examine the barriers to the uptake of cCBT from a wider range of source types that previous reviews, including the NICE guidelines	Inclusion criteria Studies of a variety of research designs and from both primary and secondary care settings on cCBT, defined as interventions where the computer took a lead in decision-making and was more than a medium. Data on acceptability, accessibility and adverse consequences were extracted Exclusion criteria –	• 36 (quantitative and qualitative studies) • Yes • Yes	• Narrative • Yes (EPOC, criteria of Mays and Pope, criteria of Crombie) • No • Professionals and staff	Barriers only
Adaji A 2008 [17] The use of information technology (IT) to enhance diabetes management in primary care: a literature review Literature review	To review the impact of IT on diabetes management in primary care and to identify the barriers and facilitators to using IT in this role	Inclusion criteria Only original studies which evaluated the use of IT interventions (web based programmes, electronic medical records, messaging systems) for diabetes management in medical practice published after 1996 in English were reviewed. RCTs or observational (non RCTs, pre-post studies, post-intervention studies) or qualitative methods Exclusion criteria Studies evaluating the use of IT for other chronic diseases, reviews papers which described other studies and commentary; studies evaluating the use of telemedicine (videoconferencing and telephone based consultations between patients and physicians)	• 29 (quantitative and qualitative) • Yes • Yes	• Unclear (narrative) • No • No • Professionals and staff	Facilitators and barriers
Fitzpatrick LAD 2008 [40] Understanding communication capacity—communication patterns and ICT usage in clinical settings Literature review	To review the literature on inter-clinician communication problems, impacts on clinical workflows, ICT usage and barriers to communication and information systems	Inclusion criteria Studies that discussed inter-clinician communication, patterns of ICT use, the effects of ICT use on workflow and/or the barriers to adopting ICTs in traditional health care settings Exclusion criteria Studies that focused on clinician-patient communication	• 98 (qualitative and quantitative studies) • Yes (no descriptions of screening process) • Yes	• Narrative • No • No • Unclear	Barriers only
Jarvis-Selinger S 2008 [56] Clinical telehealth across the disciplines: lessons learned Literature review	Key lessons learned related to programme (technology) adoption and organisational readiness	Inclusion criteria None stated	• 225 (quantitative and qualitative) • Not clearly described • Not given	• Unclear • No • No • Unclear	Facilitators and barriers
Jimison H 2008 [90] Barriers and drivers of health information technology use for the elderly, chronically ill, and underserved Systematic review	To review the evidence on the barriers and drivers to the use of interactive consumer health information technology (IT) by specific populations, namely the elderly, those with chronic conditions or disabilities, and the underserved	Inclusion criteria Studies of all designs that described the direct use of interactive consumer health IT (a consumer interacts directly with the technology, the computer processes the information in some way, a consumer receives or has access to patient-specific information in return) by at least one of the populations of interest Outcomes: technology use, health related behaviours, health service utilisation, disease status, quality of life and functional outcomes	• 52 on barriers; 60 on facilitators (qualitative and quantitative) • Yes • Yes	• Analysis based on frameworks as recommended by Popay et al. • Yes (quality rating criteria developed by the US Preventive Services Task Force and the Common Drug Review Process) • No • Not specified	Facilitators and barriers
Orwat C 2008 [84] Towards pervasive computing in health care—a literature review Literature review	To provide an overview of recent developments and implementations of pervasive computing systems in health care	Inclusion criteria Prototypes, tests, pilot studies and case studies conducted in health care settings, or systems involving prospective end users, clinical trials as well as systems already in routine use Exclusion criteria Experiments in non-medical settings as well as mere descriptions of concepts, designs or architectures	• 69 (unclear study types) • Yes • Yes	• Narrative (approach of Cruz-Correia et al. was partly adopted) • No • No • Not specified	Facilitators and barriers
Broens TH 2007 [34] Determinants of a successful telemedicine implementations: a literature study Literature review	To identify the determinants that influence the implementation of telemedicine applications	Inclusion criteria Limited to studies published after the telemed 2004 conference held in London, which they consider to be representative of telemedicine initiatives in Europe Exclusion criteria –	• Unclear • Yes • Not described	• Analysis based on the knowledge barriers categorisation of Tanriverdi and Iacono. • No • Yes (see above) • Not specified	Facilitators and barriers
Yarbrough A 2007 [43] Technology acceptance among physicians: a new take on TAM Systematic review	To look at the literature on physician acceptance of information technology	Inclusion criteria English and peer-reviewed publications only Exclusion criteria Not directly pertaining to physician IT, physician barriers to technology, the technology acceptance model Non-physician-specific technology acceptance articles, physician-specific articles, especially the users targeted were not physicians, articles attempting to create typologies of physician users. Case studies of organisations that were purely descriptive in nature and limited to less than two sites were excluded, as were review articles that only summarised findings	• 18 (quantitative and qualitative) • Yes • Yes (flowchart not given)	• Analysis based on the Technology acceptance model (TAM) • No • Yes • Professionals and staff	Facilitators and barriers
Yusof M 2007 [44] Health information systems adoption: findings from a systematic review Systematic review	To identify the most important factors of health information system adoption	Inclusion criteria Study design: case study Intervention: any computer based information systems that involves human interaction used in health care settings Exclusion criteria Study design: experimental and survey All computers or knowledge based training and education systems for professionals (not directly related to clinical care)	• 55 (quantitative and qualitative studies, e.g. documentations, questionnaire, interview, observations) (participants include managers, clerical staff, doctors and nurses) • Unclearly described • Yes	• Qualitative analysis using a theoretical framework (Human, Organisation and Technology-fit framework) • Yes (qualitative research appraisal criteria); majority—sound quality • Yes • Users including physicians and staff	Facilitators and barriers
Ohinmaa A 2006 [64] What lessons can be learned from telemedicine programmes in other countries? Literature review	To identify examples of successful telemedicine programmes	Inclusion criteria Articles that showed a scientific basis for successful telemedicine. The review focused on applications benefiting significant segments of the health care population, rather than those restricted to a targeted population or geographical area Exclusion criteria Programmes from developing countries that were seen to be difficult to implement in the US health care system; articles discussing non-medical applications	• Unclear • Unclearly described • No	• Unclear • Unclear • No • Unclear	Facilitators and barriers
Leatt P 2006 [41] IT solutions for patient safety—best practices for successful implementation in health care Narrative review	To review the literature on the facilitators and barriers to successful implementation of electronic medical records, electronic medication administration records and computerised provider order entry	Inclusion criteria Unclear	• Unclear • No • Not described	• Analysis based on framework by Klein et al. (managerial support, financial resource availability, implementation climate and implementation policies and practices) • No • Yes • Unclear	Facilitators and barriers
Peleg M 2006 [37] Decision support, knowledge representation and management in medicine Narrative review	To review the literature to find trends in CDSS that were developed over the last few decades and give some indication of future directions in developing successful, usable clinical decision support systems	Inclusion criteria Papers that were published during the past 5 years with the words Decision support systems appearing in the title and used our own knowledge of the field for earlier work	• Unclear • No • Not described	• Unclear • No • No • Unclear	Facilitators only
Shekelle P 2006 [69] Costs and benefits of health information technology Evidence report	To examine the barriers that health care providers and health care systems encounter that limit implementation of electronic health information systems	Inclusion criteria Qualitative studies that were primarily focused on barriers and studies that collected quantitative data on barriers were included Exclusion criteria Topic not about health information technology, outcomes not relevant. Studies in which barriers were briefly discussed but were not a primary focus were excluded	• 20 (quantitative and qualitative studies) • Yes • Yes	• Narrative • No • No • Professionals and staff	Barriers only
Garg AX 2005 [65] Effects of computerised clinical decision support systems on practitioner performance and patient outcomes Systematic review	To review controlled trials assessing the effects of computerised clinical decision support systems (CDSSs) and to identify study characteristics predicting benefit	Inclusion criteria Randomised and non-randomised controlled trials that evaluated the effect of a CDSS compared with care provided without a CDSS on practitioner performance or patient outcomes Exclusion criteria -	• 100 trials • Yes • Yes	• Narrative • No (not on studies of barriers/facilitators) • No • Unclear	Facilitators and barriers
Kawamoto K 2005 [73] Improving clinical practice using clinical decision support systems: a systematic review of trials to identify features critical to success Systematic review	To identify features of clinical decision support systems critical for improving clinical practice	Inclusion criteria Studies had to evaluate the ability of decision support systems to improve clinical practice. RCTs Exclusion criteria Less than 7 units of randomisation per study arm; study not in English; mandatory compliance with decision support system; lack of description of decision support content or of clinician interaction with system; and score of <5 points on a 10-point scale assessing 5 potential sources of study bias	• 70 (quantitative only) • Yes • Yes	• Descriptive and meta-regression (and frequency) analysis to identify independent predictors of success • Yes • No • Unclear	Facilitators and barriers
Lu YC 2005 [42] A review and a framework of handheld computer adoption in health care	To review the literature on issues related to adoption of Personal digital assistants (PDA) in health care and barriers to PDA adoption	Inclusion criteria Articles addressing all health care professionals and their uses of PDAs and mobile computing devices were identified Exclusion criteria –	• Unclear • Unclear • Not described	• Analysis based on the technology acceptance model • No • Yes • Professionals and staff	Facilitators and barriers
Johnson K 2001 [76] Barriers that impede the adoption of paediatric information technology Literature review	To review the literature to better elucidate barrier that are likely to affect the adoption of IT by paediatric professionals	Not stated	• Unclear • No • No	• Analysis based on framework (modified) • No • Yes (conceptual framework by Knapp: situational, cognitive, legal and attitudinal) • Physicians	Barriers only
**Preventative care and public health**					
Zheng MY 2014 [107] Physician barriers to successful implementation of US preventive services task force routine HIV testing recommendations Literature review	Focuses on physicians’ barriers to HIV testing	Inclusion criteria Literature related to HIV testing guidelines, physician adherence to HIV testing guidelines and physician barriers to HIV testing for adult primary care setting. Literature was also gathered from the HIV literature ListServ released by Dr Robert Malow, a well-known resource within the field of HIV/AIDS research Exclusion criteria Articles related to HIV testing exclusively in prenatal, paediatric, and/or emergency settings. Non-US based studies since physicians in other countries may face different and unique barriers	• Not stated (quantitative and qualitative studies) • Unclear • Not described	• See below • No (no discussion of quality of papers) • Analysed using Cabana’s model, knowledge, attitudes and behavioural skills • Physicians	Barriers only
Child S 2012 [86] Factors influencing the implementation of fall prevention programmes: a systematic review and synthesis of qualitative studies Meta-ethnography	To identify key factors that act as barriers and facilitators to the effective implementation of evidence-based best practice in relation to the prevention of falls among community-dwelling older people	Inclusion criteria Studies that examined influences on the implementation of fall prevention programmes among community-dwelling older adults and used recognised qualitative methods of data collection and analysis Exclusion criteria Editorials, opinion papers, conference abstracts	• 19 qualitative studies (6 studies—perspective of health care professionals; 12 from the experiences of community- dwelling older adults; 1 study—perspectives from both patients and health care workers in a falls clinic) • Yes • Yes	• Meta-ethnography • Yes (structured approach to describe quality by Wallace et al.) • Unclear • Health care professionals	Facilitators and barriers
Eisner D 2011 [57] Screening and prevention in Swiss primary care: a systematic review Systematic review	To identify barriers and facilitators for physicians to participate in any preventive measures	Inclusion criteria Articles that addressed screening and prevention activities in Swiss primary care. Studies which were conducted in settings in which a primary care provider played a key role were also included. Exclusion criteria No/implicit GP setting Main prevention aspects other than medical (e.g. economic)	• 49 (45 descriptive studies; 4 RCTs) Areas covered: infectious disease, lifestyle changes, cardiovascular risk factors, cancer, HIV, osteoporosis, addiction and others • Yes • Yes	• Narrative • Yes (CONSORT) (low quality in general) • No • GPs	Facilitators and barriers
Johnson M 2011 [61] Barriers and facilitators to implementing screening and brief intervention for alcohol misuse: a systematic review of qualitative evidence Systematic review	To synthesise qualitative evidence for barriers and facilitators to effective implementation of screening and brief intervention for alcohol misuse in adults and children over 10 years	Inclusion criteria Studies that addressed screening and/or brief intervention with alcohol users over the age of 10 years Exclusion criteria Studies that focused on educational interventions and school-based interventions due to their inclusion in recent UK guidance. Reports of interventions of >30 min in duration, or that were carried out by specialists	• 47 qualitative studies • Yes • Yes	• Narrative summary • Yes (source of quality checklist unknown) (very good or good quality largely) • No • Primary care teams (largely GPs and nurses)	Facilitators and barriers
Taylor CA 2011 [60] Enhancing delivery of health behaviour change interventions in primary care: a meta-synthesis of views and experiences of primary care nurses Meta-synthesis	To systematically find an synthesise qualitative studies that elicited the views and experiences of nurses involved in the delivery of HBC interventions in primary care, with a focus on how this can enhance delivery and adherence of structured HBC interventions	Inclusion criteria Studies using qualitative methods to elicit nurses’ views and experiences of delivering HBC interventions, aiming to facilitate adoption of physical activity and/or healthy eating by adult patients (age 16–65 years) within primary care. Studies were included if they utilised qualitative methods for the collection and analysis of data. This included qualitative studies as components of wider trials Exclusion criteria Not a qualitative study; intervention not delivered by nurses/does not state; not primary care	• 9 qualitative studies • Yes • Yes	• Meta-synthesis • Yes (CASP tool for qualitative research) (good quality in general) • No • Primary care nurses	Facilitators and barriers
Vedel I 2011 [70] Barriers and facilitators to breast and colorectal cancer screening of older adults in primary care: a systematic review Systematic review	To determine the barriers and facilitators to breast and colorectal cancer screening of older adults, from the perspectives of patients and primary care physicians	Inclusion criteria Studies that used a quantitative design that reported barriers and/or facilitators to CRC and breast cancer screening for older adults; the participants included physicians working in primary care and/or older adults in primary care Exclusion criteria Editorials, comments, letters, case reports, reviews, guidelines, consensus statements; studies of treatment approaches or case findings; studies assessing interventions or PCP’s actual screening performance or patient-physician communication without information on the decision-making process	• 42 (quantitative and qualitative; questionnaires and 21 on PCP’s point of view) • Yes • Yes	• Narrative • Yes (STROBE, MOOSE) • No • Primary care physicians	Facilitators and barriers
Stead M 2009 [80] Factors influencing European GPs’ engagement in smoking cessation: a multi-country literature review Literature review	To explore the extent of GPs’ engagement in smoking cessation and the factors that influence their engagement	Inclusion criteria Studies needed to report the extent to which GPs engage in smoking cessation activity or explore factors, of any sort, influencing this engagement Studies that correlated the relationship between a particular factor and their provision of smoking cessation advice. Studies that explored GP’s own perceptions of salient issues that constrained or facilitated their engagement. Qualitative and quantitative Exclusion criteria Discussion and papers that did not report original research	• 205 (100 academic and 105 grey), reporting on 188 different studies) Pre-specified categories of influencing factors: GP characteristics, patient characteristics, structural factors, and cessation-specific knowledge and skills. • Yes • Yes (flow chart not given)	• Analysis based on pre-specified categories • No • No • GPs	Facilitators and barriers
Berry JA 2008 [62] Make each patient count. Overcoming barriers to clinical preventive services Literature review	To explore barriers to wider implementation of clinical preventive services	Inclusion criteria English language studies from 1987	• Unclear • Not described • Not described	• Descriptive/narrative • No • No • Professionals (physicians and nurse practitioners)	Barriers only
Durlak JA 2008 [32] Implementation matters: a review of research on the influence of implementation on programme outcomes and the factors affecting implementation Literature review	To assess the impact of implementation on program outcomes and to identify factors affecting the implementation process	Inclusion criteria The primary focus was on prevention and health promotion programmes for children and adolescents related to the following topics: physical health and development, academic performance, drug use, and various social and mental health issues Qualitative and quantitative studies and only English language articles were included. Studies with control groups and one group pre-post designs were included. Commentaries of several authors based on their extensive research or field experiences were included Exclusion criteria None	• 81 qualitative and quantitative studies (The review also assess impact of implementation on outcomes, e.g. high vs. low implementation, well vs. poorly implemented programmes—not relevant to this review of review; not extracted) • Not described • Yes	• Analysis based on Wandermann’s framework • No • Yes (Wandersmann’s “ecological framework for understanding effective implementation) • Unclear	Factors
Hearn LA 2006 [82] Review of evidence to guide primary health care policy and practice to prevent childhood obesity Literature review	To identify key barriers to effective engagement of primary health care (PHC) providers and families in promoting healthy weight among children aged 2–6 years, and to examine promising interventions to identify policy goals to over these barriers	Inclusion criteria RCTs, process, impact, parallel and intuitive evidence were included Primary care providers included general practitioners, practice nurses, community/child/maternal health nurses, allied health professional (e.g. dieticians, physiotherapists and exercise physiologists), multicultural and indigenous health workers, and health education/promotion specialists Interventions aimed to reduce risk factors for obesity in children aged 2–6 years, focused on prevention and early intervention, were non-commercial, involved PHC providers as key facilitators of change, encouraged participation of family members, evaluated the intervention outcomes, process and/or acceptability Exclusion criteria –	• 45 (unclear study types) • Yes • Yes	• Unclear • Yes (all selected interventions were appraised and categorised as high, medium, or low standard using a scoring system with pre-set criteria (secondary appraisal to capture promising interventions), based on the method of Flynn et al.) • Yes (various theories described) • Primary health care providers	Barriers only
Nilsen P 2006 [36] Effectiveness of strategies to implement brief alcohol intervention in primary health care Systematic review	To evaluate the effectiveness of promoting brief alcohol implementation by health care providers in primary health centres and evaluates the results in relation to the implementation strategies employed	Inclusion criteria The study had to: be based on health care providers’ practices within PHC settings; include training components for physicians and/or nurses to implement brief intervention; measure the effectiveness of implementation in terms of material utilisation rate, screening rate, brief intervention rate; measure the effectiveness either before and after or only after the implementation, with or without a control group; be pragmatic (i.e. the procedures were integrated into the routine practice of the PHC office); be published in English, in a peer-reviewed scientific journal Exclusion criteria Studies that involved staff training but relied on additional on-site personnel for administering the screening of patients were not deemed naturalistic enough to warrant inclusion in this systematic review	• 11 (of which 5 are RCTs, 5 non randomised studies, 1 quasi-experimental study) • Yes • Yes	• Descriptive/narrative • No • No • Professionals	Barriers only [from discussion]
**Integration of new role**					
Sangster-Gormley E 2011 [54] Factors affecting Nurse practitioner role implementation in Canadian practice settings: an integrative review Integrated review	To review the literature about the Canadian experience with nurse practitioner role implementation and to identify influencing factors at the practice setting level	Inclusion criteria Published and unpublished Canadian NP implementation studies between 1997 and July 2010 were included Qualitative and quantitative studies of implementation or integration of the NP role in acute, primary health and long-term care settings Exclusion criteria Early studies of NP role implementation prior to legislation and regulation of the role. Role development studies were excluded. Discussion papers, theoretical papers and studies of extended or expanded nursing roles were also excluded Definition Role implementation refers to the process used to establish the NP role in a practice setting and is a component of role integration	• 10 published studies and two provincial papers (of which 5 papers are in primary care, and only these results are extracted) (quantitative and qualitative) • Yes • Yes	• Thematic analysis • No • No • Unclear	Facilitators and barriers
DiCenso A 2010 [55] Factors enabling advanced practice nursing role integration in Canada Scoping review	To develop a better understanding of advanced practice nursing role, their current use, and the individual, organisational and health system factors that influence their effective integration in the Canadian health care system	Inclusion criteria Data from the literature were synthesised from 1990 onwards, to identify enablers to role development and implementation across the different types of advanced practice nurses: clinical nurse specialists, primary health care nurse practitioners and acute care nurse practitioners	• 468 (largely primary studies, essays, editorials) • Yes (study screening/selection) • Yes (flow diagram)	• Descriptive/narrative • No (scoping review) • No • Advanced practice nursing, e.g. nurse practitioners, primary health care nurse practitioners, advanced practice nurse	Facilitators and barriers
Clarin OA 2007 [108] Strategies to overcome barriers to effective nurse practitioner and physician collaboration Systematic review	To review common barriers to effective NP and physician collaboration to identify the strategies to overcome these obstacles	Inclusion criteria English articles published within the past 10 years; published worldwide; descriptive studies showing inter-professional relationships of NPs and physicians; stories of collaboration Settings: acute care and primary practice Exclusion criteria Articles on nurses and physician collaboration and involving NP collaboration with other health care members aside from physicians	• 12 (6 based in primary care setting) (unclear study types) • No • No	• Unclear • No • No • Physicians and nurse practitioners	Barriers only
Halcomb E 2004 [85] Australian nurses in general practice based heart failure management: implications for innovative collaborative practice Narrative review	To describe the current and potential role of the practice nurse in heart failure (HF) management	Inclusion criteria Only articles which focused on the development of the practice nurse role and nursing interventions or the role of the practice nurse in the management of HF were included in the review Exclusion criteria Articles that examined the role of general practice in chronic disease management or the use of evidence-based guidelines in general practice	• 12 (survey) • No • No	• Descriptive/narrative • No (quality was discussed in the main text) • No • GPs and nurse practitioners	Facilitators and barriers
*Prescribing behaviour*					
Mason A 2008 [33] New medicines in primary care: a review of influences on general practitioner prescribing Systematic review	To explore the determinants of uptake, the causes of geographical variations and the influence of price, costs and financial incentives on prescribing behaviour	Inclusion criteria Studies need to evaluate factors affecting the uptake of new medicines in primary care; quantitative and qualitative study designs were included Exclusion criteria Not about new medicines, not about factors affecting prescribing, reviews, focused on secondary care, articles that were unobtainable	• 28 (quantitative and qualitative) • No • Yes	• Analysis based on Bonair and Persson’s framework • No • Yes • GPs	Facilitators and barriers
*Others*					
Davies SL, 2011 [109] A systematic review of integrated working between care homes and health care services Systematic review	To evaluate the different integrated approaches to health care services supporting older people in care homes, and identify barriers and facilitators to integrated working	Inclusion criteria Interventions designed to develop, promote or facilitate integrated working between care home or nursing home staff and health care practitioners. Interventions that involved staff going in to provide education/training to care home/nursing home staff were included as long as there was some description of joint working or collaboration For a study to be included there had to be evidence of at least 1 of the following: Clear evidence of joint working, joint goals or care planning, joint arrangements covering operational and strategic issues, shared or single management arrangements, joint commissioning at macro and micro levelsStudies also had to report at least one of the outcomes pre-defined in the protocol Exclusion criteria Studies where staff were employed specifically for the purpose of the research without consideration of how the findings might be integrated into ongoing practice	• 17 (10 quantitative, 1 mixed methods, 2 process evaluations, 3 qualitative, 1 action research) • Yes • Yes	• Framework analysis • Yes (Cochrane) • No • Unclear	Facilitators and barriers
Xyrichis A 2008 [110] What fosters or prevents inter-professional teamworking in primary and community care? A literature review Literature review	To explore the factors that inhibit or facilitate inter-professional teamworking in primary care and community care	Inclusion criteria Papers from non-acute health care areas such as primary care and community care, as well as from countries outside the UK. Exclusion criteria Articles not relevant with the topic under investigation, not written in English, dated prior to 1994, non-research articles and papers that were not published in accessible journals	• 10 (survey, qualitative studies) • Yes • Yes	• Thematic analysis • Yes (unclear source; limitations were discussed, per study) • No • Primary care staff	Facilitators and barriers
Baker R 2010 [63] Tailored interventions to overcome identified barriers to change: effect on professional practice and health care outcomes Cochrane review (update) Systematic review	To assess the effectiveness of interventions tailored to address identified barriers to change on professional practice or patient outcomes	Inclusion criteria RCTs that studied the effect of tailored interventions to address identified barriers (undertaken before the design and delivery of the intervention) to change on professional practice Studies had to involve a comparison that did not receive a tailored intervention (no intervention/intervention that is not tailored to identified barriers, or intervention targeted at both individual and social/organisational barriers vs. intervention target at only individual barriers) Barriers may be identified by methods including observation, focus group discussions, interviews or surveys of the involved health care professionals, and/or through analysis of the organisation/system in which care is provided	• 26 (of which 15 trials were based in primary or community care, 7 in hospital/specialist care, 3 in both, 1 in nursing home) • Yes • Yes	• Descriptive • Criteria described by EPOC for RCTs and the EPOC data collection checklist • Yes (a number of theories were described) • Unclear	Barriers only

### Methodological quality of included reviews

The level of methodological detail reported varied across reviews. Sixty-eight reviews (97 %) reported the use of explicit inclusion/exclusion criteria. Screening and data abstraction process were adequately described (e.g. independently, in duplicate, use of piloted forms, as per PRISMA checklist [21]) in 45 reviews (64 %). Thirty-nine reviews (56 %) summarised the study selection process (as a form of flow chart and/or described in the text) and the characteristics of included primary studies.

Thirty-two reviews (46 %) critically appraised their included primary studies using some form of checklist/assessment, e.g. the Critical Appraisal Skills Programme (CASP) [29] and Pluye’s mixed methods review scoring checklist [30], or described quality issues in the “Results” or “Discussion” section. Theoretical frameworks were described in 25 reviews (36 %). Many of them used theory to explain the findings in their discussion or as part of their introduction or background [3, 13, 16, 31–43]. Relatively few of the reviews used theoretical frameworks as a way to carry out their analysis [32–34, 41, 43, 44]. Examples of theories discussed in the reviews included the Diffusion of Innovations Theory [45], Normalization Process Theory (NPT) [46], the Consolidated Framework for Implementation Research (CFIR) [47], Technology Acceptance Model (TAM) [48] and the Promoting Action on Research Implementation in Health Services (PARIHS) framework [49, 50]. Further information about methodological quality (e.g. type of critical appraisal checklist or assessment form used by the included reviews) can be found in Table 1.

### Final conceptual framework

**Fig. 2:**
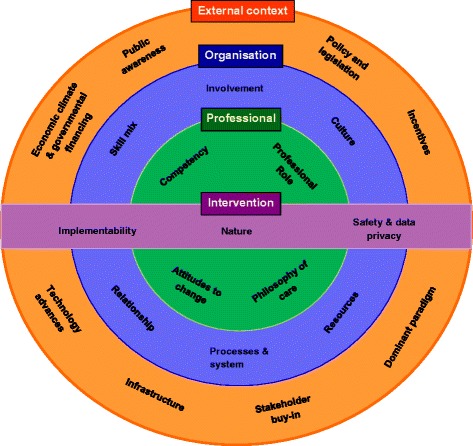
Conceptual framework describing key elements that influence implementation of change in primary care

A total of 21 primary themes and 40 secondary themes emerged from the data and were classified into the four levels of *external context*, *organisation*, *professionals* and *intervention*, described in detail below. Examples of quotations from the included reviews are provided to illustrate themes in Table 2. Many reviews mentioned the dynamic relationships among factors as an important issue in implementation. However, almost all reviews presented individual barriers and/or facilitators as separate concepts without exploring how the barriers and facilitators interacted or their relative importance. Overall, there was a lack of information about the context in which the different barriers and facilitators occurred. All the themes drawn from the identified reviews were treated equally and attributed the same weight independent of their frequency to avoid problems arising from potential “double counting” of primary studies included in several reviews. The final conceptual framework describing the different levels is presented in Fig. 2.

**Table 2:** Key themes related to the success or failure of implementation of complex interventions

	Primary themes	Secondary themes	Sources	Example quotations from included reviews	Domain					
					G	M	E	PU	I	PR
External context	Policy	Presence and form of policy	[14, 15, 31, 34, 43, 51–55, 58, 86, 101]	B: A lack of a national mandate within countries to coordinate fall prevention interventions [86] F: Legislative mandates are also potent motivators [51]	√	√	√	√	√	
		Presence of stated goals and objectives	[41, 57, 67]	B: Lack of clear national objectives [57] F: Convey a clear statement of the goals for and anticipated benefits of electronic medical records implementation [41]		√	√	√		
		Fit with local or national agenda	[16, 32, 55]	B/F: Compatibility (contextual appropriateness, fit, congruence, match)—extent to which the intervention fits with an organisation’s mission, priorities and values [32]		√		√	√	
		Presence of regulatory framework	[41, 51, 53, 54, 56, 58, 65, 67, 85, 94]	B: Restrictive regulatory framework [54] F: Federal mandates and a common framework that provides standards and procedures that allow systems to exchange information, regardless of whether both support highly coded data [58]		√	√		√	
		Presence of code of practice	[34, 41, 43, 51, 58, 61, 67, 85]	F: New practice standards, guidelines and routines must be established for how work gets done [41]		√	√	√	√	
	Infrastructure		[14, 15, 42, 53, 58, 67, 72, 74, 75, 82, 86, 101, 104]	B: Inadequate employment contracts, practice facilities and functioning of the primary care team [85] F: Mechanism of support and infrastructure to support health care professionals [85]	√	√	√	√	√	
	Economic and financing		[53–55, 58, 66, 67]	B: Lack of investment by health authorities [66]	√	√	√		√	
	Incentives	Financial awards	[3, 14–16, 31, 33, 35, 37, 39, 43, 51, 53, 56–59, 61–63, 66, 68–70, 74, 77, 79, 81, 83–86, 89, 90, 101, 103, 104, 107, 110]	B: No financial gain in using evidence-based medicine [66] F: Other incentive schemes include quality and outcomes framework, which offers incentive payments linked to several prescribing targets; risk-sharing schemes [33]	√	√	√	√	√	√
		Non-financial awards	[14–16, 31, 53, 59–61, 64, 68, 74, 89]	B: Lack of incentives to change practice [68] F: Access to training are important incentives for general practitioners [61]	√	√	√	√	√	
	Dominant paradigm		[15, 16, 33, 54, 63, 77, 86, 94]	B/F: NICE (The National Institute for Health and Care Excellence) and other guidelines [33]	√	√	√	√	√	√
	Public awareness		[34, 55, 67, 105]	B: Inadequate public awareness of advanced practice nursing roles [55] F: Widespread dissemination is important to create awareness among stakeholders, either by impersonal channels or mass media, to motivate the introduction and usage of telemedicine [34]		√	√		√	
	Stakeholder buy-in		[15, 16, 31, 41, 42, 44, 53–55, 57, 60, 64, 94, 103]	B: Conflict potential: Lack of consensus, decision power, and commitment among key stakeholders. It includes the inadequate distribution of decision-making power (or ownership) among stakeholders [94] F: Board members are aligned with implementation plan [16]	√	√	√	√	√	
	Technological advances		[65, 67]	B/F: Those responsible for Clinical Decision Support System implementation are typically administrators, information technology managers, and clinicians, all of whom are increasingly pushed by technology [65]		√	√			
Organisation	Culture	Organisational planning and readiness	[3, 14, 15, 32, 34, 38, 40, 41, 51, 54–56, 58, 60, 61, 65–67, 69, 71, 77, 83, 94, 103, 105, 109, 110]	B/F: Receptiveness of the whole organisation [56]	√	√	√	√	√	
		Leadership	[14–16, 31, 32, 34, 41, 52–56, 58, 65, 67–69, 71, 75, 94, 103, 109, 110]	B: Lack of organisational, nursing and physician leadership and support frequently reported as a barrier to role implementation for all types of advanced practice nurse roles [55]	√	√	√	√	√	
		Hierarchy structure	[44, 54, 67]	B/F: Hierarchical structure in the setting [54]		√	√		√	
	Processes and systems		[14, 17, 31, 32, 34, 37, 39, 40, 42–44, 51, 52, 57, 59, 65, 66, 69, 71–74, 77, 84, 90, 105]	B: Even when the practitioners have access, guidelines are often insufficiently integrated into current behavioural, organisational and communication routines [52] F: Process—Work process was the most important factor of this theme (24 elements). When e-prescribing was integrated, work process was facilitated and work flow was improved [14]	√	√	√	√		
	Relationships	Inter-professional	[3, 14–16, 31–33, 41, 53–55, 60, 67, 71, 72, 75, 86, 105, 108, 110]	B/F: The organisational aspect of professional interaction, including team spirit, relation between different health professionals [14]	√	√	√	√	√	√
		Professional and patients	[31, 41, 58, 61, 72, 75, 78]	B/F: Interaction: patient-physician encounters [58]	√	√	√	√		
	Resources		[3, 14, 16, 17, 31–36, 38–41, 43, 51–57, 59, 60, 62–72, 75–77, 79, 80, 82, 84–87, 90, 94, 106, 107, 109]	B: The lack of resources such as time, money and personnel constitutes a significant barrier [71] F: Administrative support, adequate resources and manpower, dedicated or protected time [67]	√	√	√	√	√	√
	Skill mix	Clarity about responsibility/role	[14, 16, 32, 44, 54, 55, 58, 61, 67, 71, 72, 85, 110]	B: Lack of clarity pertaining to the responsibility inherent in the role of care manager (often a nurse) when it comes to promoting the patient’s self-management ability [71] F: Procedures that contain clear roles and responsibilities relative to task accomplishments [32]		√	√	√		
		Division of labour	[34, 41, 44, 51, 55, 56, 58, 60, 61, 67, 71, 77, 79, 95, 103, 110]	B: Lack of organisation and skill mix among support staff [67] F: Different skill mix (interdisciplinary approach) [77]		√	√	√	√	
	Involvement	Support from team members and management	[14, 31, 32, 39, 41, 54, 55, 58, 60, 61, 66, 67, 90, 104, 105, 109, 110]	B: Lack of managerial support [61] F: Organisational support and management [31]	√	√	√	√	√	
		Collaborative working	[14–16, 32, 34, 41, 54, 55, 67, 71, 72, 78, 82, 94, 104, 109]	B: Lack of team approach to change [16] F: Collaborative process is characterised by non-hierarchical relationships among participants, mutual trust and open communication, shared responsibilities for competing important tasks and efforts to reach consensus when disagreements arise [32]	√	√	√	√	√	
		Shared vision	[16, 32, 41, 54, 55, 60, 67, 71, 72, 77, 110]	B/F: Shared vision (shared mission, consensus, commitment, staff buy-in)—extent to which organisational members are united regarding the value and purpose of the innovation [32]		√	√	√	√	
Professional	Role	Professionalism	[14, 16, 31, 35, 39, 53–55, 65–67, 72, 85, 89, 102, 110]	B: Fear of loss of autonomy [35] F: General practitioners provided practice nurses with considerable autonomy in managing clients with chronic conditions with defined practice guidelines and protocols [85]	√	√	√	√	√	
		Sense of self-efficacy	[14–16, 32, 33, 38, 39, 61, 63, 68, 78, 79, 101, 104, 107, 109]	B/F: Sense of self-efficacy [15]	√	√	√	√		√
		Peer influences	[14, 31, 38, 66]	B/F: The opinion/attitudes of colleagues about evidence-based medicine [66]	√		√			
		Authority/influence	[33, 38, 67, 101, 103]	B: “Not having enough authority to change patient care procedures” (nurses) [38]	√	√				√
	Underlying philosophy of care	Personal style	[42, 54, 61, 69, 72, 76, 78, 79, 105, 107]	B/F: Physician personality and philosophy [54]		√	√	√	√	
		Relationship between professional and patient	[3, 14, 33, 39, 42, 60, 62, 77–80, 107]	B/F: Perception of inconsistency of recommendations with patient values and preferences [3]	√	√	√	√		√
	Attitudes to change	Attitudes and beliefs (general)	[3, 14–17, 31–36, 38–41, 43, 44, 52, 57, 58, 60–72, 75–80, 82, 83, 85, 87, 89, 90, 100–102, 105–109]	B: Staff attitudes to advanced care planning have adversely affected uptake [101] F: Agreement with the particular information and communication technologies (general attitude) [31]	√	√	√	√	√	√
		Motivation and priority	[31, 36, 39, 42, 57, 59, 61–63, 67, 68, 70, 82, 107]	B: Physicians may not have the motivation to change. Results suggest that close to half of physicians surveyed were in a pre-contemplation stage and not ready to change behaviour [39]	√	√	√	√		
		Prior experience	[15, 34, 54, 61, 66, 67, 77, 107]	B/F: Users’ previous experiences with health information system affected their experience with a new system both positively and negatively [77]	√	√	√	√	√	
		Workload/competing demands	[16, 17, 31, 44, 54, 55, 61, 66, 67, 71, 72, 74, 77, 79, 84, 105]	B: As the professionals seemed overburdened with papers and administrative tasks, they had difficulty allocating time to help people with depression [71]	√	√	√	√	√	
		Perception of time	[3, 14–17, 31, 36, 38–40, 43, 44, 61, 62, 66, 70, 72, 74, 75, 77, 79, 83, 86, 90, 101, 107]	B: “Having insufficient time on the job to implement new ideas” (nurses) [38] F: Saves clinicians time or requires minimal time to use [74]	√	√	√	√		
	Competencies		[3, 14–17, 31–36, 38–43, 51, 54, 56–58, 60–62, 66–70, 72, 75–80, 82, 84–87, 90, 100, 101, 105–109][55]	B: Non-existent or inadequate training [31] F: Electronic medical record (EMR) implementation was found to be most effective when training for EMR system users was adequate, timely, tailored to meet the specific needs and experience of the users and available on an ongoing, as-needed basis [41]	√	√	√	√	√	√
Intervention	Nature and characteristics	Complexity	[3, 16, 35, 37, 39, 67, 68, 70, 72, 87–89]	B: Confusing and complex recommendations [3] F: Not overly complex [68]	√	√	√	√		
		Evidence of benefit	[3, 16, 31, 33, 34, 38, 39, 42, 43, 51, 55, 56, 59, 62, 63, 67, 68, 70, 74–76, 85, 89, 103, 106, 107]	B: Lack of evidence regarding benefits of Information Technology [43] F: Improved quality of care, e.g. better health outcomes, reduce medical errors [51]	√	√	√	√	√	√
		Applicability and relevance	[14, 16, 31, 34, 35, 37–39, 44, 55, 66–69, 82, 89, 94, 105]	B: Evidence has a limited scope/focus or limited to particular populations [68]	√	√	√	√	√	
		Clarity	[4, 9, 18, 19, 26, 35, 42, 52, 59, 101]	B: Uncertainty about when to initiate advanced care planning discussions—timing [101] F: Good clarity [35]	√	√	√		√	
		Costs	[16, 84, 94]	B: Generating indicators is costly [16]		√	√			
		Cost-effectiveness	[33, 34, 42, 51, 57, 58, 65, 67, 69]	B: Cost-effectiveness relation perceived as unfavourable [57] F: Improved cost-effectiveness and efficiency [67]		√	√	√		√
		Practicality and utility	[3, 14–16, 31, 34, 35, 37, 39–42, 44, 52, 56–58, 65–70, 72–74, 77, 82, 84, 87, 89, 91, 94, 101]	B/F: Ease of use of the system [68]	√	√	√	√		
		Adaptability	[15, 32, 34, 77, 94]	B/F: Adaptability of interventions to local circumstances (program modification, reinvention, flexibility), extent to which the proposed program can be modified to fit provider preferences, organisational practices, and community needs, values, and cultural norms [32]	√		√			
		IT compatibility	[14, 16, 31, 42, 56, 67–69, 71, 73]	B: Interoperability—Inadequate interfacing with other IT systems [14] F: IT is current or resources available for upgrading [16]	√	√	√			
	Implementability	Complexity of implementation	[31, 57, 68]	B: Too complex project organisation [57] F: Do not require a great deal of time or effort to implement [68]	√		√	√		
		Benefit/harm of implementation	[14, 41, 43, 51, 56, 58, 76]	B: Implementation results in lower provider productivity and inconsistent error reduction [43] F: More efficient workflow, e.g. less time spent handling lab results, improved access to clinical data, streamlined referral processes, reduced staff time [51]			√			
		Resources requirements	[15, 34, 42, 43, 56, 68, 71, 77]	B: Too costly to implement [68]	√	√	√			
	Safety and data privacy		[3, 14, 31, 33, 34, 39–43, 51, 55, 56, 67, 69, 70, 73, 76, 77, 84, 90, 101, 105–107]	B: Concerns over data protection and security [106] F: Benefit of anonymity for sensitive health topics [90]	√	√	√	√	√	√

*B* barriers, *F* facilitators *G* guideline, *M* management of care, *E* e-health, *PU* public health and preventative medicine, *I* integration of new roles, *PR* prescribing

### External context

*Policy and legislation*. The presence of supportive national and local policies which were mandatory and appropriate legislative mechanisms often acted as potent activators [51] and promoted implementation of clinical guidelines, telemedicine and new roles (e.g. nurse practitioners) [34, 52–56]. Secondary themes associated with policy and legislation included *fit with local or national agenda*, where compatibility between interventions and local or national policies, or an organisation’s mission, priorities and values promoted adoption [16, 32]. Conversely, the lack of *stated goals and objectives* reflecting priorities and directions could act as barriers [57]. Similarly, the *regulatory framework*, particularly where it was restrictive or absent was found to impede implementation [41, 51, 58]. *Presence of codes of practice*: having standards (usually at the local level) to ensure quality and uniform practice and establishing guidelines for how work gets done were shown to promote implementation.

The presence of clear *incentivisation* structures was found to drive adoption: this included *non-financial incentives* such as public recognition [59] and access to training [60, 61]. *Financial incentives* were shown to facilitate adoption, e.g. governmental incentives for use of health information technology, quality and outcome frameworks that linked to targets for prescribing [33]. There were concerns from professionals about the lack of finance to incentivise adoption of new processes or interventions [62]. Financial penalties could lead to distrust and professional demoralisation [3].

*Dominant paradigms* refer to the presence of commonly held set of values or beliefs in a society at a given time, e.g. evidence-based practice and patient centred care. Professional organisations such as those producing national guidance and advice to improve health care and the political agenda impact upon the credibility and enactment of these commonly held values [33]. Another example included the advocacy of certain drugs by pharmaceutical industries [63].

*Buy-in by internal or external stakeholders* at different levels promoted implementation through multidisciplinary effort and by having stakeholders aligned with an implementation plan [16]. Conversely lack of stakeholder buy-in [64], resistance or competing priorities or lack of interest from stakeholders was found to impede implementation [54]. *Infrastructure:* short comings, from unreliable internet access, lack of access to information, lack of mechanisms or systems to support storing or documenting information or lack of infrastructure support for implementation were all reported to impede implementation, whereas presence of these features promoted implementation.

*Advances in technology* in health care have become increasingly salient. Technologies change health care delivery and the way in which information is provided (e.g. electronic patient records, telemedicine). There is a growth of interest in their use [65], and this is shown to drive implementation. *Economics and financing* including the economic climate, the ways in which the government allocated funding, and investment decisions made by local health authorities were shown to affect implementation of guidelines [66] and new roles [54, 55]. Finally, *public awareness* could result in pressure to introduce a new intervention. This was presented as a facilitator for motivating the uptake of telemedicine and for educating the public about new nurse practitioner roles [34, 55].

### Organisation

The presence of a positive *culture* which was receptive to change and valued innovation was viewed as important for implementation [32, 41, 58]. Strong and consistent internal and external *leadership* including identifying influential champions who were respected and trusted by staff to drive change and implementation and communicate vision, from the beginning of the project had a positive impact on adoption. Conversely, lack of effective leadership to advocate change, set priorities or manage the implementation process; and changes in leadership were presented as barriers [55, 67–69]. *Organisational readiness* is defined as the degree of preparation before implementation: lack of staff preparation or strategic planning (e.g. resource planning, implementation plan) was found to be a barrier which could be influenced by the practice environment (e.g. small practice size, inadequate practice organisation). A *hierarchical structure*, defined as the degree to which the organisation prescribed roles or responsibilities and/or promoted autonomy was mainly presented as a barrier [44, 54, 67].

*Available resources*, including time, funding, staff and technical support, were commonly reported as both barriers and facilitators. Limited funding in general, lack of time to plan [16] or train staff [51], insufficient equipment [3, 70] or administrative support to perform additional data entry or deal with paperwork [31, 67, 71, 72] were reported as barriers. Other related barriers included failure to adequately anticipate the amount of time and costs, including operational and training costs [34, 35, 44], costs associated with ongoing maintenance [34, 41] and the amount of technical assistance and support required at all stages of the project, e.g. some resources available to support the project at the beginning but insufficient for its completion [67].

*Processes and systems* were defined as the extent to which the intervention fitted with existing workflow and how well it integrated with current working processes and systems. When the fit was good [14, 73, 74], for example, when e-prescribing was sufficiently integrated as part of clinician workflow, work process was improved [14]. Achieving good fit sometimes required redesign of delivery systems or workflow.

*Relationships*, both *between professionals and between professionals and patients* were found to influence implementation. Positive and trusting *inter-professional relationships* through the presence of bi-directional communication and giving staff abundant opportunity to discuss salient matters and provide input to challenges before and during implementation were perceived to be facilitators [32, 41, 55]. Conflict with patient expectations [75] and concerns about patient and health professional interaction, for example, when using the new information system, nurses spent more time on documentation than on direct care [31], could lead to a decrease in acceptability of an intervention and subsequently impede implementation.

*Skill mix issues*, including *clarity of role and responsibility* and *division of labour*, were presented as both barriers and facilitators. A lack of clarity about accountability leading to confusion about who should be responsible for implementing the changes could constitute a barrier [76]. For instance, in relation to e-prescribing, clinicians did not want to solve implementation problems and believed this should be done by non-clinical staff [14]. The nature of the division of labour, defined as the allocation of responsibilities and the appropriate use of skills to accommodate new processes or implementation was also a factor that emerged from some reviews. The absence of personnel with the right combination of skillset or a lack of appropriate expertise to perform specific tasks (e.g. business and medical personnel with the informatics expertise to develop strategic plans for health information exchange or electronic sharing of health related information) [44, 67] were found to impede implementation. By contrast, flexibility of skill mix incorporating an interdisciplinary approach was shown to facilitate implementation. Non-clinical staff often had better knowledge of optimising processes compared to clinicians [77], and different members of the workforce brought different perspectives and skills to the implementation [77].

*Involvement*—*support from team members and management*; *collaborative working* and *shared vision*. Support from peers, colleagues and superiors, active engagement of both clinical and non-clinical staff, continuous communication from senior management about the importance of change and its consistent commitment were shown to facilitate implementation [32, 41, 60]. A team-based partnership approach, collaborative efforts and good coordination between stakeholders and organisations were all shown to be important for implementation [34, 41, 55, 77]. Collaborative processes can be characterised as being facilitated by non-hierarchical relationships, mutual respect or trust and open communication among individuals, as well as shared decision-making to determine how the intervention can or should be implemented and the ability to reach consensus when there is disagreement [32]. Shared vision, defined as a collective understanding and agreement on goals, importance and benefits of the intervention and mutually held realistic expectations about the work required for implementation, for instance, a collective understanding of resources required for implementing change and that cost savings might not occur in the short term, due to decreased productivity during implementation, was presented as both a facilitator and a barrier [16, 32, 41, 54, 77].

### Professionals

Themes within this level included perceptions of what it meant to be a professional—*professionalism, peer influence*, *sense of self-efficacy and authority/influence. Professionalism*, which included using professional judgement to apply scientific and experiential knowledge and dealing with uncertainty, was viewed as a salient aspect to be considered in relation to implementation. Concerns about reduced autonomy or trust, independence of practice or inability to practise to full scope were all shown to impede implementation [16, 31, 35, 39, 54, 55]. *Peer influences*, for example, negative attitudes or beliefs of colleagues towards information and communication technology were perceived as barriers to implementing the intervention. Moreover, a lack of confidence in one’s own ability to carry out specific tasks and the feeling of not having authority or enough influence to change or carry out the procedures were found to impede implementation [31, 66].

*Underlying philosophy of care* includes *personal style* and *relationship between health professionals and patients*. Personal style, defined as the perceived fit between the intervention and the preferred style of clinical practice, such as clinicians’ communication style [78, 79], personality [54] and philosophical opposition to the intervention [69, 76], were presented exclusively as barriers. Additionally, patient values and preferences [3, 33] and concerns about clinician-patient relationships [42, 77] impeded implementation (e.g. concerns that new systems would affect clinician-patient relationship).

*Attitudes to change, prior experience, motivation and priority, familiarity and awareness, perception of time and workload.* Attitudes and beliefs are shaped by personal beliefs and experience, education and training and peer networks. This was perceived as an important aspect to consider in relation to implementation and was reported as both a barrier and a facilitator. Resistance to change caused by disagreement with the evidence, negative beliefs about the usefulness or added value of the intervention or belief that the intervention was not part of their role were commonly described as a barrier to implementation [3, 14, 35, 41, 67–70, 76, 77, 80]. Previous personal experience in clinical practice or with the information system affected professional attitudes to a new system or intervention [54, 66, 77, 81]. Competing priorities [42, 57, 59, 62, 63, 70, 82], lack of motivation [31, 39, 42, 57, 68, 81] and low awareness of the intervention [31, 35, 38, 39, 43, 44, 68, 75, 80, 81] were shown to impede implementation. Further, perceived shortage of time, for example, to plan or implement new ideas, to carry out new interventions or procedures or to learn new skills, were commonly presented as a barriers in the included reviews [3, 17, 31, 36, 38–40, 43, 62, 70, 75, 79, 81, 83]. Additional workload caused by the implementation of new complex interventions was also found to hinder adoption [17, 31, 44, 54, 66, 81, 84].

Lastly, *competencies*, e.g. adequate training and good computer experience/skills were shown to facilitate implementation [16, 17, 31, 32, 34, 41, 42, 44, 54–58, 60, 67, 70, 77, 80, 81, 84–86].

### Intervention

*The nature and characteristics* of the intervention which included the *complexity of the intervention*, *evidence of benefit*, *applicability and relevance*, *costs of an intervention*, *cost-effectiveness of an intervention*, *clarity*, *practicality and utility of intervention*, *customisation of intervention and IT compatibility* were all viewed as aspects to be considered during implementation. Interventions that were complex were often associated with lower adoption [3, 35, 39, 87, 88]. By contrast, interventions that demonstrated clear and consistent clinical evidence of benefit [3, 16, 34, 35, 51, 55, 56, 68, 74, 89] or good applicability relevant to setting [3, 35, 68] were shown to facilitate implementation. The costs of an intervention and whether practices could obtain a positive return on investment [51] and in particular the time invested [42, 51] were considered to be features that would promote implementation. A lack of cost-effectiveness evidence relevant to the setting or poor cost-effectiveness could impede implementation [33, 57]. Additionally, interventions with good definitional clarity, such as well-organised guidelines with well-defined measurable actions that were based on clear strong recommendations, promoted implementation [35, 68]. Complex interventions that demonstrated good design, e.g. an overview of patient information (current health status and patient history) with a follow-up of patient adherence to their prescription and access to laboratory results was a facilitator of implementing e-prescribing [14] and showed good usability and reliability, e.g. user-friendly, easily accessible, fast, provides accurate and up-to-date information, content relevant to user, automatic prompting, information given at the time of decision-making [31, 35, 58, 69, 73, 74, 90] were associated with successful implementation. *Customisation of intervention*—the degree to which a new intervention can be modified to make it more applicable to specific contexts was a relevant factor. New interventions that could be customised to fit provider needs and preferences, organisational practices, values and cultural norms were shown to promote implementation [32, 37]. In addition, interventions compatible with the current operating IT system were more likely to be implemented [16, 56].

*Implementability* included the *complexity of implementation process*, *benefit and harm as a result of implementation and resource requirement*. The *complexity of implementation* can be determined by the scale of implementation, number of sites and processes required. Highly complex implementation plans were less likely to succeed as they often required complex project organisation [57]. Effective project management (e.g. using an incremental approach over time according to a strategic plan allowing a transition period between old and new system) was shown to facilitate implementation [77]. *Benefit and harm as a result of implementation*—adoption of a new intervention or process might bring potential benefit or harm to other aspects of care. For instance, implementing a new intervention usually required shifting organisational priorities and putting other projects on hold which resulted in initial lower productivity and increased staff workload [41, 76]. Conversely, implementation of a new intervention might lead to cost savings or more efficient workflow [51, 58]. *Resources required for implementation*—effective implementation required sufficient resources and funding to support not only start-up costs but also on-going costs and attention to sustainability [56].

Finally, *safety and data privacy* were perceived to be important for implementation. With regard to technology based interventions, there were concerns from both health care professionals and patients about the ownership of health information, secure data exchange, unauthorised sharing of confidential information about patients with fear of discrimination based on the health condition [51] and liability if patient information was lost [41]. The presence of sufficient security mechanisms to support trust between providers and patients [34] and technical measures to ensure systems compliance with data protection laws [84] were shown to promote implementation.

### “Fit” between the intervention and the context

Our conceptual framework has highlighted the importance of the “fit” between the intervention and the different levels of context (Fig. 2), i.e. external context, organisation and professionals; how well the intervention fits with the external context (e.g. current policy, national or local agenda, existing infrastructure) and whether the organisation’s existing work practices (e.g. culture, readiness, relationships and leadership) and daily work as well as their beliefs and values (professional attributes), will have an impact on the degree of implementation and intervention outcomes. This hypothesis requires empirical testing.

### Dynamic nature of barriers and facilitators

The literature suggested that relationships between individual barriers and facilitators are subject to change over time. This was rarely described in great detail. A review of electronic prescribing implementation found a change in individuals’ perceptions between the different stages of implementation. While the users had a less positive view of the intervention during pre-implementation phase, their views became more positive with the increasing use of the intervention during the transition and post-implementation phases. In addition, work processes were viewed as a barrier during the transition phase but became a facilitator when the intervention was formally integrated and work flow was improved [14].

### Relevance of contextual factors according to different topic domains/complex interventions

Table 2 shows which contextual factors are related to different complex interventions/topic domains. Dominant paradigm (commonly held set of values or beliefs in a society at a given time), financial incentives, resources, competencies, attitudes to change (in general), inter-professional relationships, evidence of benefit, and safety, confidentiality and liability concerns were common implementation considerations. Wider contextual issues such as policy, infrastructure and organisational culture (except inter-professional relationships) were not perceived as issues relevant to changing prescribing behaviour. Most contextual factors were perceived to be relevant to implementation of E-health technology. Whilst it is useful to know what the likely barriers or facilitators are for implementing certain types of complex interventions, these findings need to be interpreted with caution. The findings might highlight the barriers likely to arise during implementation; however, contextual factors need to be considered as a whole as every organisation is unique and thus may be more or less affected by particular contextual issues.

## Discussion

In this systematic review of reviews, we sought to identify the causes, or given explanations or influences operating in the evidence-practice gap, relating to the implementation of complex interventions. We could not examine “causes” of the evidence to practice gap due to the absence of data, as well as the nature of the reviews, particularly the way their analyses were carried out: they mostly used a descriptive approach by reporting individual barriers and facilitators without stating the relationships between them. There is also a lack of information about the context in which these barriers and facilitators occur. A large number of multi-level contextual influences emerged from the included reviews related to the levels of external context, organisation, professionals and intervention. This review has demonstrated the challenges associated with implementation and that implementing any type of change in primary care is likely to be complex. Our conceptual framework has been developed based on published reviews of studies with empirical evidence from different types of complex interventions and topic domains. Its development was different from other existing frameworks or models such as the Consolidated Framework for Implementation Research (CFIR) and the Normalization Process Theory (NPT). CFIR was developed using a meta-theoretical approach, combining constructs across published theories or frameworks [47]. NPT was constructed from a sociological perspective [91, 92] and the Theoretical Domains Framework (TDF) which is an integrative framework of theories of behaviour change developed using an expert consensus process [93]. Despite taking a different methodological approach, the content of our framework (data derived from primary care) is comparable and overlaps considerably with the CFIR which is not primary care specific and has resonance with NPT. This has enhanced the validity of our findings.

Relevant barriers and facilitators are dynamic and likely to change over time [14, 28]. Despite many of the barriers being reported as separate entities in the identified reviews, they are likely to interact with one another and each cannot be considered in isolation [28, 32, 47, 94]. This finding is consistent with the systematic review undertaken by Greenhalgh et al., i.e. many studies failed to address important interactions between different levels and account for contextual and contingent issues [95]. Contextual factors that are perceived as barriers at the beginning of implementation may become facilitators later on in the implementation process [14].

### The importance of context in implementation in primary care

This review has highlighted the importance of paying attention to context which is often notably absent from research and frequently fails to be acknowledged, described or taken into account during implementation. It is unclear how it can be described, defined and measured [96]. Bate et al. suggested that context can be studied using mixed methods (e.g. participatory observations, interviews, documentary analysis), in order to get a richer picture of how different contextual factors influence implementation [96]. Other methods such as contextualisation and context theorising have been proposed to address the multi-level and dynamic nature of context [96]. The updated Medical Research Council (MRC) guidance for process evaluation of complex interventions stresses the relevance of taking into account the contextual factors associated with variations in implementation, intervention mechanisms and outcomes [97]. Our review suggests the need to pay attention to the external context as well as the specific context within which a complex intervention is being embedded.

### Strengths and limitations of study

To the best of our knowledge, this is the first systematic review of reviews that provides a comprehensive overview related to the field of implementation in primary care. This review is not restricted to any type of clinical topic or discipline. The broad scope is a strength as we aimed to produce a single document which summarises and synthesises the literature that is easily accessible to clinicians, researchers and policy makers. A key advantage of undertaking a systematic review of reviews is the ability to summarise and synthesise a vast and fragmented literature relatively efficiently. It enables synthesis at different levels, allowing comparisons across different complex interventions, different outcomes and different health conditions or population groups. We could not determine the relative “weighting” of the findings as it would not be appropriate: this is a review of reviews and not of primary studies. Furthermore, this is a qualitative synthesis which focuses on not only the primary studies but also the authors’ interpretations derived from these studies. Our analysis has accounted for all the themes emerged from the included reviews.

Despite our attempt to be as inclusive and comprehensive as possible, the search may not have identified all relevant literature; this risk has been minimised by screening reference lists of all included papers for additional literature. Equally, the reviews included in this article may not have captured all the primary research studies; therefore, some findings may be missed. However, we are confident that this is unlikely to change the conclusions of this review. In addition, formal quality assessment was not undertaken and this could be a potential weakness of the study. Nevertheless, the papers included had to meet the criteria of “review” using the definitions by Mair et al. [13], and an attempt was made to describe and summarise the quality of the reviews using PRISMA. Double coding was only undertaken in a proportion of the included reviews. However, we took a rigorous and cyclic approach through every step in our data synthesis (i.e. extensive involvement and discussions among all the authors in reviewing the extracted data and refinement of concepts at every stage: from pilot synthesis, construction of descriptors and extraction grid, to translations synthesis and the final conceptual framework). Furthermore, the importance of using methods of validation (i.e. use of multiple coders, assessment of inter-rater reliability) and their applicability to qualitative research/evidence synthesis is less clear and controversial [98, 99].

A major limitation is the conceptualisation of factors affecting the second translational gap as “barriers” and “facilitators”. A study exploring the value of “barriers to change” suggested that barriers were constructions used by the participants to make sense of the situation in which they found themselves and implementation studies must look beyond the narrative that is provided by participants [28]. Most original studies included in the reviews are surveys or of accounts of research participants through qualitative interviews or focus groups. Perceptions of barriers may be socially constructed, and addressing them may not necessarily improve implementation [79]. In our work, we could only analyse and report data from included reviews, and despite our initial question focusing on the causes of the evidence to practice gap, the overwhelming dominance of the use of the framework of “barriers and facilitators” required us to also adopt this framework to report on existing data.

## Conclusions

We took a multi-level approach to synthesise data from 70 reviews, addressing barriers and facilitators to implementation of complex interventions in primary care. This resulted in the development of a conceptual framework which emphasised the importance and inter-dependence of (1) the external context in which implementation was taking place; (2) organisational features; (3) characteristics of health professionals involved and (4) characteristics of the intervention. Understanding the context, the interplay between facilitators and barriers to implementation and considering the “fit” between the intervention and the context are likely to be essential in determining the degree to which implementation of any one intervention is successful. This evidence-based conceptual framework could be used by health care researchers and/or primary care organisations that seek to improve uptake of effective complex interventions, by identifying and overcoming their context-specific issues.

### Implications for research

Despite the identification of a large number of reviews and many topics being discussed, there are gaps in the literature. Studies beyond barriers and facilitators are required and a more explanatory approach (how and why) should be used. Future research needs to focus on articulating how and why each contextual factor is important in influencing the uptake of a particular intervention. In addition, we need to describe the interactions between these contextual influences and understand their relative importance. A more theoretically driven approach may help with understanding, describing, defining and potentially measuring context.

### Implications for clinical practice and policy

**Fig. 3:**
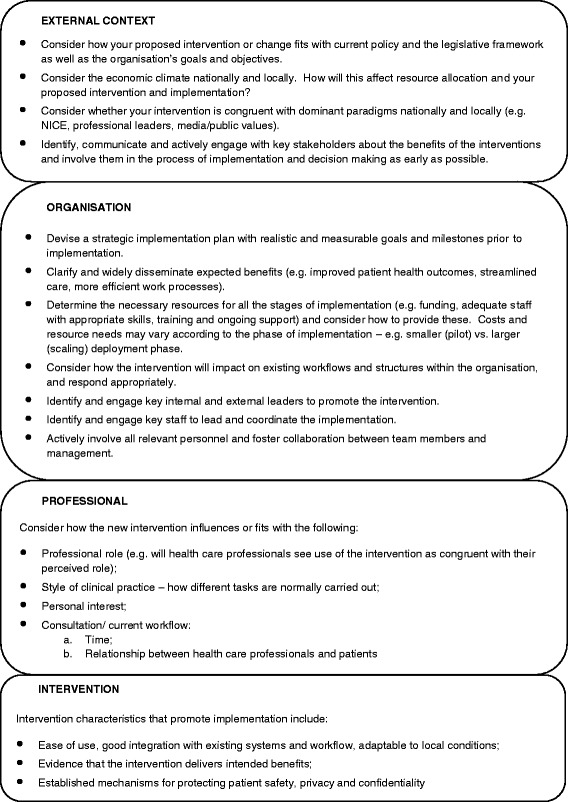
Practical implications of the results of the synthesis—recommendations when planning implementation

Implementation of any type of intervention is complex, dynamic and influenced by a variety of factors at the level of external context, organisation, professional and intervention in the primary care setting. Understanding and defining context appeared to be important and the “fit” between the intervention and the context has been highlighted. A list of recommendations was constructed from the review findings and can be found in Fig. 3. Individuals who wish to implement any type of change in their organisation should (1) consider and describe the context they are working in and (2) monitor context periodically as it is likely to change over time.

## Additional files

Additional file 1: Medline search. Literature search strategy used in MEDLINE. (DOCX 19 kb)

Additional file 2: Scope of the review-domains and types of complex interventions included in the review. Broad and specific topic domains included in the review, e.g. guidelines on various topics, different types of ehealth interventions. (DOC 29 kb)

Additional file 3: ENTREQ statement checklist. ENTREQ reporting checklist to enhance transparency in reporting the synthesis of qualitative research. (DOC 43 kb)

## Abbreviations

CFIR: Consolidated Framework for Implementation Research
MRC: Medical Research Council
NHS: National Health Service
NPT: Normalization Process Theory
TDF: Theoretical Domains Framework
USA: United States of America
