# Supplementary material for: Achieving change in primary care—causes of the evidence to practice gap: systematic reviews of reviews
Source: Implement Sci. 2016 Mar 22;11:40. doi: 10.1186/s13012-016-0396-4 (PMC4802575; doi:10.1186/s13012-016-0396-4)
Supplement: Supplementary file 1 — Medline search. Literature search strategy used in MEDLINE. (DOCX 19 kb) [file 13012_2016_396_MOESM1_ESM.docx]

**Additional files**

Additional file 1 – MEDLINE search strategy

1 Translational Medical Research/

2 translational gap.mp.

3 knowledge transfer.mp.

4 research uptake.mp.

5 knowledge translation.mp.

6 evidence to practice.mp.

7 evidence practice gap.mp.

8 research practice gap.mp. [mp=title, abstract, original title, name of substance word, subject heading word, protocol supplementary concept, rare disease supplementary concept, unique identifier]

9 exp Evidence-Based Practice/

10 research to practice.mp.

11 Guideline Adherence/

12 1 or 3 or 4 or 5 or 6 or 10 or 11

13 2 or 7 or 8

14 primary care.mp.

15 exp General Practice/

16 general practi*.mp. [mp=title, abstract, original title, name of substance word, subject heading word, protocol supplementary concept, rare disease supplementary concept, unique identifier]

17 GP.mp. [mp=title, abstract, original title, name of substance word, subject heading word, protocol supplementary concept, rare disease supplementary concept, unique identifier]

18 general practitioners/ or physicians, family/ or physicians, primary care/

19 Nurse Practitioners/ or Primary Care Nursing/

20 family doctor*.mp.

21 family practice.mp. [mp=title, abstract, original title, name of substance word, subject heading word, protocol supplementary concept, rare disease supplementary concept, unique identifier]

22 primary medical care.mp. [mp=title, abstract, original title, name of substance word, subject heading word, protocol supplementary concept, rare disease supplementary concept, unique identifier]

23 family medicine.mp. [mp=title, abstract, original title, name of substance word, subject heading word, protocol supplementary concept, rare disease supplementary concept, unique identifier]

24 family physician*.mp. [mp=title, abstract, original title, name of substance word, subject heading word, protocol supplementary concept, rare disease supplementary concept, unique identifier]

25 primary health care/ or "continuity of patient care"/

26 14 or 15 or 16 or 17 or 18 or 19 or 20 or 21 or 22 or 23 or 24 or 25

27 12 and 26

28 systematic review.mp.

29 meta-synthesis.mp.

30 meta-ethnography.mp.

31 narrative review.mp.

32 "Review"/

33 Meta-Analysis/

34 "Review Literature as Topic"/

35 Qualitative Research/

36 28 or 29 or 30 or 31 or 32 or 33 or 34

37 meta.mp.

38 35 and 37

39 36 or 38

40 implement*.mp.

41 integrat*.mp.

42 adopt*.mp.

43 normali*.mp.

44 facilitat*.mp.

45 routini*.mp. [mp=title, abstract, original title, name of substance word, subject heading word, protocol supplementary concept, rare disease supplementary concept, unique identifier]

46 diffusion.mp. [mp=title, abstract, original title, name of substance word, subject heading word, protocol supplementary concept, rare disease supplementary concept, unique identifier]

47 dissemination.mp. [mp=title, abstract, original title, name of substance word, subject heading word, protocol supplementary concept, rare disease supplementary concept, unique identifier]

48 gap.mp.

49 barrier*.mp.

50 obstacle*.mp. [mp=title, abstract, original title, name of substance word, subject heading word, protocol supplementary concept, rare disease supplementary concept, unique identifier]

51 cause*.mp. [mp=title, abstract, original title, name of substance word, subject heading word, protocol supplementary concept, rare disease supplementary concept, unique identifier]

52 promotor*.mp. [mp=title, abstract, original title, name of substance word, subject heading word, protocol supplementary concept, rare disease supplementary concept, unique identifier]

53 48 or 49 or 50 or 51 or 52

54 40 or 41 or 42 or 43 or 44 or 45 or 46 or 47

55 27 and 39

56 53 or 54

57 9 and 26 and 39 and 56

58 29 or 30

59 26 and 58

60 13 and 39

61 intervention*.mp. [mp=title, abstract, original title, name of substance word, subject heading word, protocol supplementary concept, rare disease supplementary concept, unique identifier]

62 Medical Records Systems, Computerized/ or Electronic Health Records/

63 Telemedicine/

64 Decision Making, Computer-Assisted/ or Decision Support Systems, Clinical/

65 Medical Informatics/

66 Models, Organizational/

67 Organizational Innovation/

68 61 or 62 or 63 or 64 or 65 or 66 or 67

69 26 and 39 and 54 and 68

70 56 and 58

71 53 and 54

72 26 and 39 and 71

73 55 or 57 or 59 or 60 or 69 or 70 or 72
