# Supplementary material for: Achieving change in primary care—causes of the evidence to practice gap: systematic reviews of reviews
Source: Implement Sci. 2016 Mar 22;11:40. doi: 10.1186/s13012-016-0396-4 (PMC4802575; doi:10.1186/s13012-016-0396-4)
Supplement: Supplementary file 2 — Scope of the review-domains and types of complex interventions included in the review. Broad and specific topic domains included in the review, e.g. guidelines on various topics, different types of ehealth interventions. (DOC 29 kb) [file 13012_2016_396_MOESM2_ESM.doc]

Additional file 2 Scope of the review – domains and types of complex interventions included in the review

| Broad topic* | Specific topics covered in the review |
| --- | --- |
| Guidelines or evidence-based practice | Guidelines in general, children and adolescent mental health, arthritis, chronic diseases, children with attention-deficit-hyperactivity disorder, in rural and remote practice. |
| E-health | Computerised decision support system, computerised cognitive behavioural therapy, electronic prescribing, electronic medical records, information and communication technologies or health information exchange, e-health service in rural communities, telemedicine, telehealth, paediatric information technology |
| Management of care | Chronic care model/ chronic diseases, advanced care planning in palliative care, process/quality improvement, quality measurement, audit, mental health, dementia, depression, diabetes, nurse-led care |
| Public health or preventative medicine | HIV testing, fall prevention programmes, breast and colorectal cancer screening, behaviour change interventions, brief alcohol interventions, smoking cessation |
| Integration of new role or collaborative working | Nurse practitioner role implementation, nurse-physician collaboration, collaborative practice or inter-professional team working |
| Prescribing | Change in prescribing practice/behaviour |

**Topic domains are not mutually exclusive.*
