# Supplementary material for: Achieving change in primary care—causes of the evidence to practice gap: systematic reviews of reviews
Source: Implement Sci. 2016 Mar 22;11:40. doi: 10.1186/s13012-016-0396-4 (PMC4802575; doi:10.1186/s13012-016-0396-4)
Supplement: Supplementary file 3 — ENTREQ statement checklist. ENTREQ reporting checklist to enhance transparency in reporting the synthesis of qualitative research. (DOC 43 kb) [file 13012_2016_396_MOESM3_ESM.doc]

Additional file 3 The ENTREQ statement

| No. | Item | Description | Completed on page # |
| --- | --- | --- | --- |
| 1 | Aim | State the research question the synthesis addresses. | ✔(page 6) |
| 2 | Synthesis methodology | Identify the synthesis methodology or theoretical framework which underpins the synthesis, and describe the rationale for choice of methodology (e.g. meta-ethnography, thematic synthesis, critical interpretive synthesis, grounded theory synthesis, realist synthesis, meta-aggregation, meta-study, framework synthesis). | ✔(page 10) |
| 3 | Approach to searching | Indicate whether the search was pre-planned (comprehensive search strategies to seek all available studies) or iterative (to seek all available concepts until they theoretical saturation is achieved). | ✔ pre-planned (page 7) |
| 4 | Inclusion criteria | Specify the inclusion/exclusion criteria(e.g. in terms of population, language, year limits, type of publication, study type). | ✔(page 7) |
| 5 | Data sources | Describe the information sources used (e.g.electronic databases (MEDLINE, EMBASE, CINAHL, psycINFO, Econlit), grey literature databases (digital thesis, policy reports), relevant organisational websites, experts, information specialists, generic web searches (Google Scholar) hand searching, reference lists) and when the searches conducted; provide the rationale for using the data sources. | ✔(page 7) |
| 6 | Electronic search strategy | Describe the literature search (e.g. provide electronic search strategies with population terms, clinical or health topic terms, experiential or social phenomena related terms, filters for qualitative research, and search limits). | ✔(additional file 1) |
| 7 | Study screening methods | Describe the process of study screening and sifting (e.g. title, abstract and full text review, number of independent reviewers who screened studies). | ✔(page 8 and 9) |
| 8 | Study characteristics | Present the characteristics of the included studies (e.g. year of publication, country, population, number of participants, data  collection, methodology, analysis, research questions). | ✔(page 9 and 12, table 1) |
| 9 | Study selection results | Identify the number of studies screened and provide reasons for study exclusion (e,g. for comprehensive searching, provide numbers of studies screened and reasons for exclusion indicated in a figure/flowchart; for iterative searching describe reasons for study exclusion and inclusion based on modifications to the research question and/or contribution to theory development). | ✔(page 9 and 12) |
| 10 | Rationale for appraisal | Describe the rationale and approach used to appraise the included studies or selected findings (e.g. assessment of conduct (validity and robustness), assessment of reporting (transparency), assessment of content and utility of the findings). | ✔(page 10) |
| 11 | Appraisal items | State the tools, frameworks and criteria used to appraise the studies or selected findings (e.g. Existing tools: CASP, QARI, COREQ, Mays and Pope; reviewer developed tools; describe the domains assessed: research team, study design, data analysis and interpretations, reporting). | ✔key items of quality chosen as per PRISMA and based on the experience of the authors (page 10) |
| 12 | Appraisal process | Indicate whether the appraisal was conducted independently by more than one reviewer and if consensus was required. | ✔(page 10) |
| 13 | Appraisal results | Present results of the quality assessment and indicate which articles, if any, were weighted/excluded based on the assessment and give the rationale. | ✔(page 13-14) |
| 14 | Data extraction | Indicate which sections of the primary studies were analysed and how were the data extracted from the primary studies?  (e.g. all text under the headings “results /conclusions” were extracted electronically and entered into a computer software). | ✔(page 10) |
| 15 | Software | State the computer software used, if any. | ✔ Excel spreadsheet |
| 16 | Number of reviewers | Identify who was involved in coding and analysis. | ✔(page 11) |
| 17 | Coding | Describe the process for coding of data (e.g. line by line coding to search for concepts). | ✔(page 10-12) |
| 18 | Study comparison | Describe how were comparisons made within and across studies  (e.g. subsequent studies were coded into pre-existing  concepts, and new concepts were created when deemed necessary). | ✔(page 11) |
| 19 | Derivation of themes | Explain whether the process of deriving the themes or constructs was inductive or deductive. | ✔(page 10) |
| 20 | Quotations | Provide quotations from the primary studies to illustrate themes/constructs, and identify whether the quotations were participant quotations of the author’s interpretation. | ✔(page 10, 14 and table 2) |
| 21 | Synthesis output | Present rich, compelling and useful results that go beyond a summary of the primary studies (e.g. new interpretation, models of evidence, conceptual models, analytical framework, development of a new theory or construct). | ✔(figure 2, 3 and discussion) |
